# Dual transcriptional-translational cascade permits cellular level tuneable expression control

**DOI:** 10.1093/nar/gkv912

**Published:** 2015-09-23

**Authors:** Rosa Morra, Jayendra Shankar, Christopher J. Robinson, Samantha Halliwell, Lisa Butler, Mathew Upton, Sam Hay, Jason Micklefield, Neil Dixon

**Affiliations:** 1 Manchester Institute of Biotechnology, University of Manchester, Manchester, M13 9PL, UK; 2 Faculty of Life Sciences, University of Manchester, Manchester, M13 9PL, UK; 3 School of Chemistry, University of Manchester, Manchester, M13 9PL, UK; 4 School of Biomedical & Healthcare Sciences, Plymouth University Peninsula Schools of Medicine and Dentistry, Plymouth, PL4 8AA, UK; 5 SYNBIOCHEM, University of Manchester, Manchester, M13 9PL, UK

## Abstract

The ability to induce gene expression in a small molecule dependent manner has led to many applications in target discovery, functional elucidation and bio-production. To date these applications have relied on a limited set of protein-based control mechanisms operating at the level of transcription initiation. The discovery, design and reengineering of riboswitches offer an alternative means by which to control gene expression. Here we report the development and characterization of a novel tunable recombinant expression system, termed *RiboTite*, which operates at both the transcriptional and translational level. Using standard inducible promoters and orthogonal riboswitches, a multi-layered modular genetic control circuit was developed to control the expression of both bacteriophage T7 RNA polymerase and recombinant gene(s) of interest. The system was benchmarked against a number of commonly used *E. coli* expression systems, and shows tight basal control, precise analogue tunability of gene expression at the cellular level, dose-dependent regulation of protein production rates over extended growth periods and enhanced cell viability. This novel system expands the number of *E. coli* expression systems for use in recombinant protein production and represents a major performance enhancement over and above the most widely used expression systems.

## INTRODUCTION

Most inducible bacterial recombinant expression systems operate at the transcriptional level. An example is the *E. coli* BL21(DE3) strain, which utilizes the T7 bacteriophage RNA polymerase (T7 RNAP) in combination with expression vectors containing the T7 promoter. This system is widely used despite the well-studied issues of leaky basal expression and heterogeneous induction response (1). Inducible microbial recombinant expression systems often display either an all-or-none response with limited titratability, or a heterogeneous response to induction within a host cell population (2,3). This can be troublesome for the production of technically challenging proteins, where, e.g. aggregation, toxicity and/or solubility are an issue (1). Furthermore, high levels of recombinant expression are known to lead to over utilization of cellular resources, metabolic burden, ribosome destruction and compromised cell viability (4,5). In addition to the effects of the recombinant system upon the cell, cell physiology and stress can affect the functionality (6) and stability (7) of recombinant systems. Various methods to alleviate some of these limitations have been attempted, either at the transcriptional level, e.g. by use of tight promoters (1), or at the protein level, e.g. by allosteric repression/regulation of T7 RNAP (8,9). However, further development work is required to overcome many of these issues, for a range of recombinant protein production applications, metabolic engineering and synthetic biology efforts. Enabling expression to be matched to the cellular processing capacity, and/or toxicity thresholds would be optimal to avoid a waste of cellular resource allocation, and to maximize titre and yield of the target protein of interest. To permit matching of expression to capacity, inducible recombinant expression should ideally be regulated over a broad dynamic range, and with a robust analogue (tuneable) dose-response. By definition analogue dose-response curves are less sensitive to: (i) local fluctuations in activators (inducers) due to differential uptake/diffusion, (ii) metabolic heterogeneity resulting in fluctuations in catabolite levels, and (iii) transcriptional bursts and stochastic fluctuations in basal expression (10). Further from an operator level an analogue response is more robust and less sensitive to small errors in the manual addition of inducers, and thus permits greater precision and reproducibility.

Nature has developed a wide range of post-transcriptional control mechanisms by which to regulate gene expression, including: tRNA sensing (11,12), ncRNA (13), riboswitches (14), viral pseudoknots (15), ribosome pausing (16) and allosteric protein inhibition (8). Taking a lead from nature we identified the naturally occurring metabolite-responsive riboswitches as promising gene regulatory devices (14). Riboswitches are mostly found in the 5′ UTR of bacterial mRNAs, where they have been shown to operate via a number of mechanisms, including translation initiation, transcription termination or mRNA cleavage mechanisms (17–19). Upon binding to a specific metabolite, riboswitches change conformation, permitting differential gene regulation to occur (17). This is a fundamentally different regulatory paradigm from that widely used in inducible promoter recombinant systems, therefore riboswitches present themselves as attractive targets for use as novel genetic control elements (7). Previously, we re-engineered the Add-A translational ON riboswitch to no longer bind to its cognate metabolite, but instead to a synthetic inducer (20). Further, we demonstrated that the orthogonal riboswitch can control heterologous gene expression *in vivo* (20,21).

Here using both an inducible promoter and the previously developed orthogonal riboswitch (20), we developed a dual transcriptional-translational ON device to directly control the expression of both T7 RNAP, and the recombinant gene of interest in an *E. coli* host. This recombinant expression system, termed *RiboTite*, has been benchmarked against a number of the commonly used *E. coli* expression systems and strains (BL21(DE3), BL21(DE3)pLysS, LEMO21(DE3), KRX and pBAD) with the eGFP reporter gene, using in-depth biochemical and mathematical performance analysis. In addition the potential of the novel expression system has been demonstrated with the toxic gene *sacB*, the antimicrobial peptide epidermicin NI01, which is toxic to *E. coli* during recombinant expression, and clinically important targets (scFv, IFNα2a, p53, MTH1).

## MATERIALS AND METHODS

All cells were grown in TB (2.7% yeast extract, 4.5% glycerol, 1.3% Bactotryptone) or LB medium (0.5% yeast extract, 0.5% NaCl, 1.0% Bactotryptone), supplemented with 0.2% glucose. A Biotek Synergy HT Microplate Reader was used to measure the GFP fluorescence and OD_600_ for intact cells.

### Strains, plasmids, kits, inducers

BL21(DE3) (*Novagen*), BL21 parental (*Bioline*), LEMO21(DE3) (*NEB*), pLysS (*Novagen*), KRX (*Promega*), pBAD (*Life Technologies*) pMOD3, EzTn5 kit (*Cambio*). pET15b-*eGFP*, pET15-*sacB* and genes for: *scFv, IFNa2a, MTH1, p53* (*GeneArt*). IPTG (Isopropyl β-D-1-thiogalactopyranoside) (*Sigma*), rhamnose (*Sigma*), arabinose (*Sigma*), PPDA - Pyrimido[4,5-d]pyrimidine‐2,4‐diamine (*Peakdale Molecular*). Ampicillin (*Sigma*), Chloramphenicol (*Sigma*).

### Primers

1f: AAAAACCATGGCCACGATTAACATCGCTAAGAACGAC
1r: TTTTGCGGCCGCTTACGCGAACGCGAAGTCCGAC
2f: AAAGAATTCACACCATCGAATGGTGCAAA
2r: TTTGAATTCGGTGCCTAATGAGTGAGCTA
3f: ATCCGAATGATATGGTTTCG
3r: TCCATGCCGAGAGTGATCCC

### Strain and vector engineering

*T7 RNAP* was PCR amplified (p1f/r), restriction digested and ligated into the pMOD3-M6″-sp*DsRED* plasmid (previously assembled using the reported method (21)), via *Nco*I/*Not*I to afford pMOST [pMOD3-*lac_WT_* P/O-ORS-sp*T7RNAP*]. Note that M6″ and ORS (orthogonal riboswitch) are equivalent. To permit the subsequent transfer of the construct into a variety of genetic locations, with both different vector backbones and stable chromosomal insertions, we sought to provide the system with a proximal copy of the transcriptional repressor gene (*lacI*). *lacI* was amplified (p2f/r) and cloned into the pMOST vector (*Eco*RI site) to afford clones pMLOSTf and pMLOSTr, with the *lacI* gene upstream from the ORS-*T7RNAP* system in both forwards and reverse directions, respectively.

To permit greater flexibility, and to avoid the cellular burden of maintaining two plasmids, we sought to develop a strain with a stable chromosomal insertion of the functional constructs. Firstly, the pMLOSTf construct was linearized with flanking IS sites by restriction digestion (*Psh*AI), then using the EZ-Tn5 transposon system (*Epicentre*). The parental BL21 strain was transformed with the transposons, and selected for with the *kan^R^* marker. Transformants were confirmed by colony PCR, and rescue cloning into a *pir*^+^ strain confirmed both a single insertion per cell and the chromosomal location (DMSO reductase gene) of the insertion, in the resultant strain named BL21(IL3). The strain was used for subsequent analyses. The strains BL21(DE3) and BL21(IL3) provide an ideal system to compare the (+/−) effect of the riboswitch control of T7 RNAP, as the genetic backgrounds are essentially identical, and the T7 RNAP regulatory inserts contain similar design elements.

The ORS was amplified (p3f/r) and cloned into the plasmid pET15b-*eGFP* (*Xba*I/*Nco*I sites), to create the plasmid pETORS′-*eGFP*. The *Nco*I site in this plasmid was subsequently removed by site-directed mutagenesis (Qiagen), to establish the correct nucleotide sequence between the RBS and AUG start codon, this afforded the plasmid pETORS-*eGFP*. The plasmid pETORS-*eGFP* was transformed into the BL21 (DE3) and BL21 (IL3) strains. BL21 (DE3) and BL21 (IL3) bearing the pETORS-*eGFP* plasmid showed one and two layers of control respectively in a classical P_T7_ driven expression system. A series of synthetic genes (GENEART) were cloned into pET15b-*eGFP* (a kind gift from Tim Eyres, Manchester), and pETORS-*eGFP* expression vectors, using the *NdeI*/*Bam*HI restriction enzyme sites. The sequences of *IFN 2α* and *scFv* were a kind gift from Cobra Biologics. Epidermicin NI01 (22) was sub-cloned from pET28_NI01 (22) into pETORS via the *NcoI* and *BlpI* sites. eGFP was sub-cloned into the pBAD vector via the *XbaI* and *BlpI* sites.

### Expression induction procedure

LB media pre-cultures were inoculated directly from freshly plated single colonies for each of the systems, grown for 16 h, and diluted 1:100 in TB + 0.2% glucose with appropriate antibiotics, and growth was monitored at 37°C with shaking. For the multiwell scale, 400 μl cultures in early-logarithmic phase (∼0.3 OD_600_) were transferred to deep-well plates, containing a matrix of inducer concentrations and incubated at 30°C with shaking at 1000 rpm (Stuart microtitre plate shaker incubator). For Ultra-Yield (UY) flasks scale 30 ml of cultures in early-logarithmic phase (∼0.8 OD_600_) were transferred to 125 ml UY flasks containing the appropriate combination of inducers. Induced cultures were grown at 20, 30 or 37°C in a shaking incubator (210 rpm) for up to 20 h.

### Expression analysis and quantification

Cells were harvested and washed twice in PBS/0.2% tween. Relative fluorescence units (RFU) and OD_600_ were measured by multimode plate reader. RFU/OD were plotted to compare the effect of the various combinations of inducer concentrations. For SDS-PAGE and western blot analysis, OD-normalized volumes were collected and re-suspended in SDS-PAGE loading buffer. Equal volumes were loaded, separated by SDS-PAGE, and target proteins confirmed by western blot. Membranes were blocked with 5% skimmed milk in PBS/0.2% tween. eGFP was detected with mouse monoclonal Anti-His antibody (Pierce 1:3000 in 5% skimmed milk) and donkey anti-mouse HRP-conjugated secondary antibody (1:10 000 in 5% skimmed milk). Anti-RNA polymerase σ^70^ (*Pierce* 2G10) was used as a loading control. After incubation with HRP substrate, chemiluminescence was captured on film, and signal intensity analysed by densitometry (ImageJ software). Two calibration curves were constructed: (i) Western blot signal intensity versus purified eGFP standard (70–500 ng); (ii) RFU from a selection of experimental samples versus ng protein (converted using calibration curve (i)). The full experimental data set was converted from RFU to ng protein using calibration curve (ii).

### Data processing and statistical analysis

Normalized fluorescence measurements (RFU/OD) were exported from the microplate reader datasheets into Origin (OriginLab). Each data point was the mean of at least two biological experimental repeats (n ≥ 2), and was used to create Bivariate-dose gene expression matrices, or for curve fitting using a four-parameter logistic function. Error bars show calculated standard errors.

### FACS

*E. coli* BL21 (DE3) pET15b-*eGFP* and *E. coli* BL21 (IL3) pETORS-*eGFP* were grown to early-logarithmic phase and induced overnight at 30°C with 50 μM IPTG (DE3 strains) and 50 μM IPTG with varying PPDA concentration (IL3 strains). Induced cells were washed once in PBS then diluted 1/1000 in PBS. Flow Cytometry was performed using a Sony SH800 Cell Sorter (Sony Biotechnology) equipped with a 488 nm laser, enabling excitation of eGFP, with subsequent emission being measured through use of the FL2 Channel (525/50 nm). Data were recorded for 100 000 cells per sample at ≤5000 events s^−1^ and analysed using FCS Express 4 Flow Cytometry (De Novo Software). Histograms of events versus eGFP fluorescence were plotted and used to determine mean fluorescence and coefficients of variation (CV) for the total population and gated subpopulations consisting of non-fluorescent and fluorescent cells. Additionally the percentage of total events falling within each gated sub-population was recorded. In the case of overlaid histograms, the FCS Express for Flow cytometry smoothing function was applied at factor 2, to allow easy visualization of individual lines.

### SacB assay

Overnight cultures (diluted 1:100) were grown at 37°C, and inducers were added (IPTG 50μM and 200 μM PPDA) as required at OD_600_ = 0.7; the cells were then grown for a further 3 h. 10 μl drops of serial diluted (1:10) cultures were spotted on agar plates containing the inducers as required, in the presence and absence of 5% sucrose. Colony forming was observed after incubation overnight at 37°C. The colonies were counted and normalized for OD = 1.0, to calculate the normalized colony forming unity (CFU/ml).

### Preparation of SDS samples for soluble/insoluble fraction analysis

Samples were collected after 3 and 20 h of induction, and equivalent number of cells were pelleted down and re-suspended in 0.3 ml of lysis buffer (20 mM Tris pH 8500 mM NaCl, 0.2 mg/ml lysozyme) supplemented with DNAseI (NEB) and protease inhibitors (Roche). The samples were incubated on ice for 30’, sonicated, and then were spun down at 14 000 rpm for 10’ at 4°C. The supernatant was collected (soluble fraction), whereas the pellet was washed twice in PBS and then re-suspended in 0.3 ml of PBS (insoluble fraction). After adding equal volume of 2xSDS-PAGE loading buffer, the fractions were boiled for 10’ at 100°C and ran on a 4–20% SDS-PAGE gel (NuPAGE).

### LDH assay

LDH Cytotoxicity Assay was performed according the manufactory instruction (*Pierce*). The data are shown as a percent of LDH activity (A_490–630_) from cell-free media relative to the activity from chemically lysed cells.

### Clinical protein expression

Overnight cultures (diluted 1:100) were grown in UY flasks and inducers were added (IPTG 50 μM with various concentrations of PPDA) as required at OD_600_ = 0.8. Most of the cultures were grown at 37°C in a shaker-incubator for 3 h after induction. Only for ScFv gene expression, the cells were grown at 30°C for 3 or 20 h. Protein expression was detected by standard western blot procedure, using the antibodies described in *Expression analysis and quantification* method.

### Epidermicin quantitation

Expression levels were determined by quantitation of stain-free SDS-PAGE in-gel fluorescence (Bio-Rad), band intensity was normalized to total lane intensity and quantitated using the Image J software.

## RESULTS

### Translational control over T7 RNAP

**Figure 1.**
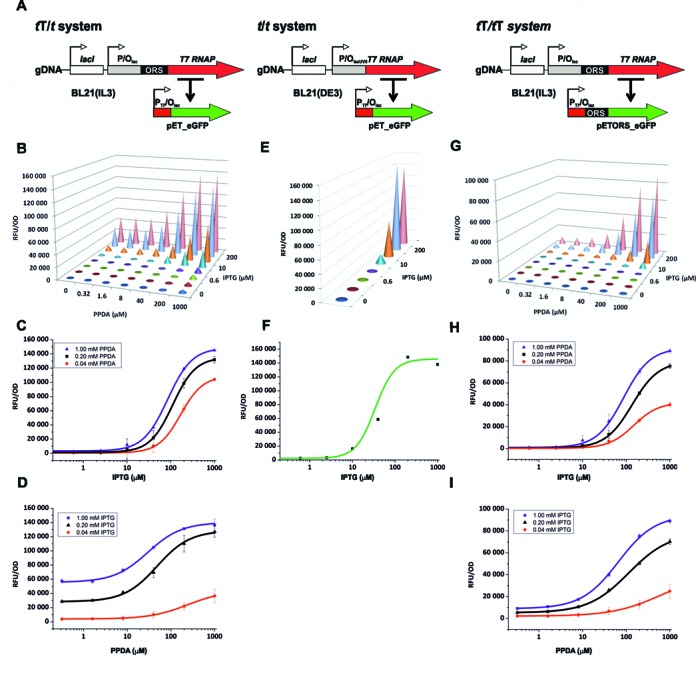
Construct Design and Multi-variant expression analysis. (**A**) the orthogonal riboswitch (ORS)-containing BL21(IL3) and standard BL21(DE3) *E. coli* expression strains, with complementary expression plasmids pET and pETORS bearing the eGFP gene. (**B–I**) eGFP expression matrices and dose response curves, for relative fluorescent units (RFU) normalized to cell density (OD_600_) under different inducer concentrations at 30° C, 3 h post induction for the systems: *t*T*/t* (**B–D**), *t/t* (**E–F**) and *t*T/*t*T (**G–I**). Dose response curves were fitted to a four-parameter logistic function. The data represent the mean of at least two replicates with error bars showing standard errors.

We wished to deploy the Pyrimido[4,5‐d]pyrimidine‐2,4‐diamine (PPDA) inducible orthogonal riboswitch (ORS) (23) in conjunction with the IPTG inducible *lac* promoter/operator (P/O*_lac_*), to achieve dual transcriptional and translational control over T7 RNAP expression. We placed the ORS between a P/O*_lac_* and the T7 RNAP gene, so that this enzyme requires induction by both IPTG and PPDA to be expressed effectively; this construct was inserted into the chromosome of the parental BL21 strain (Figure 1A, Materials and Methods). This strain, termed BL21(IL3), should effectively act as a Boolean AND logic gate, as it requires two inputs (IPTG and PPDA) to produce an output (T7 RNAP). The strain was transformed with a plasmid (pET-eGFP) containing the *eGFP* gene controlled by T7 RNAP promoter/*lac* operator hybrid (P*_T7_*/O*_lac_*), to generate a system exhibiting transcriptional (*t*) and translational (T) control over T7 RNAP, and transcriptional control (*t*) over the *eGFP* gene, named *t*T*/t*. Bivariate-dose gene expression analysis revealed dose-dependent eGFP expression in response to both inducers (Figure 1B). Furthermore, regulatory control levels (max/basal) were high at both 3 h (>400-fold), and 20 h post induction (56-fold) (Supplementary Figure S1 and Table S1). These large induction ratios were attainable due to the tight control over basal expression in the absence of induction.

By fitting the dose-response data with a four-parameter logistic function, both the half-maximal effective concentration (EC_50_), and the dynamic range of ligand response (DRLR; EC_90_/EC_10_) were determined. For IPTG titration the *t*T*/t* system demonstrated intermediate digital-analogue (tuneable expression) response profiles (DRLR *ca*. 15), independently of PPDA (Supplementary Table S2). Increasing PPDA (fixed) resulted in a vertical extension of the IPTG response curves, with little effect observed upon basal expression (Figure 1C) (24). In contrast, for PPDA titration the system exhibited classical analogue behaviour (DRLR = 79) at IPTG 40 μM, and a DRLR *ca*. 50 at IPTG ≥ 200 μM (Supplementary Table S2). In addition, EC_50_ values were dramatically affected and an increase in both basal and maximal expression levels was observed (Figure 1D). This tuning of the PPDA response at different IPTG concentrations allows for both vertical and horizontal scaling of the PPDA dose response curves, where the basal and maximal expression levels can be changed, and also the window of PPDA sensing can be modestly expanded or contracted.

### Benchmarking standard transcriptional control

To evaluate the performance of the ORS control over T7 RNAP in the *t*T/*t* system, we used the BL21(DE3) strain, that expresses T7 RNAP from the *lac_uv5_* P/O*_lac_*, and transformed it with the pET-eGFP plasmid (Figure 1A). This system with just transcriptional control over the *T7 RNAP* and *eGFP* genes was named *t/t* (Figure 1A). IPTG induced eGFP expression in a dose-dependent manner (Figure 1E), with a maximal expression output similar to the *t*T/*t* system; however, basal expression in the absence of IPTG was much higher, affording a modest 65-fold induction (Supplementary Table S1). This basal expression increased dramatically with time affording only *ca*. 3-fold after 20 h. In this instance, basal expression is the result of leaky transcription from P/O*_lac_* in the absence of IPTG. PPDA had no effect on these cells, either in terms of *eGFP* expression or cell growth (data not shown). The DRLR covered just a 10-fold range of IPTG concentration, indicative of a digital (ON/OFF) response (Figure 1F and Supplementary Table S2) (25).

### Dual orthogonal riboswitch control

To achieve greater regulatory control, we additionally placed the ORS between the T7 promoter and the *eGFP* gene, replacing the original 5′ UTR, to create the pETORS-eGFP plasmid (Materials and Methods). The BL21(IL3) strain was transformed with this plasmid, creating an expression system that combines dual input transcriptional-translational control over T7 RNAP expression, with a further dual transcriptional-translational control over the target gene output (Figure 1A). This *t*T/*t*T system offers exceptional dose-dependent response to both inputs (Figure 1G), with *ca*. 850-fold induction maximum observed 3 h post induction, and with an impressive *ca*. 330-fold induction maximum observed after 20 h, compared to 65- and 3-fold control of the *t/t* system (Supplementary Table S1). The maximal eGFP expression output was reduced to *ca*. 70% compared to the *t/t* system, however basal expression for the *t*T/*t*T system was especially low representing just *ca*. 0.1% of the maximal expression level.

For IPTG titration both the EC_50_ (87–149 μM) and DRLR values (18–22-fold) are only modestly influenced by PPDA at the fixed concentrations tested (Figure 1H). Here essentially by adjusting solely the maximal output, without affecting the basal expression level, the additional translational control acts as a rheostat, by acting as an adjustable point of resistance through the regulatory cascade. When fully induced by IPTG (1 mM), the PPDA EC_50_ was 66 μM with a 70-fold DRLR (Figure 1I), and these values responded to IPTG with inversely proportional behaviour, allowing for horizontal scaling of the dose response curves. At low IPTG concentrations (40 μM IPTG) the analogue behaviour was most pronounced with the DRLR covering a 245-fold range of PPDA concentration (Supplementary Table S2). As with the *t*T/*t* system, both vertical and horizontal scaling of the PPDA dose response curves is observed, and additionally here the *t*T/*t*T system allows the window of PPDA sensing to be greatly expanded or contracted.

### Global performance modelling

**Figure 2.**
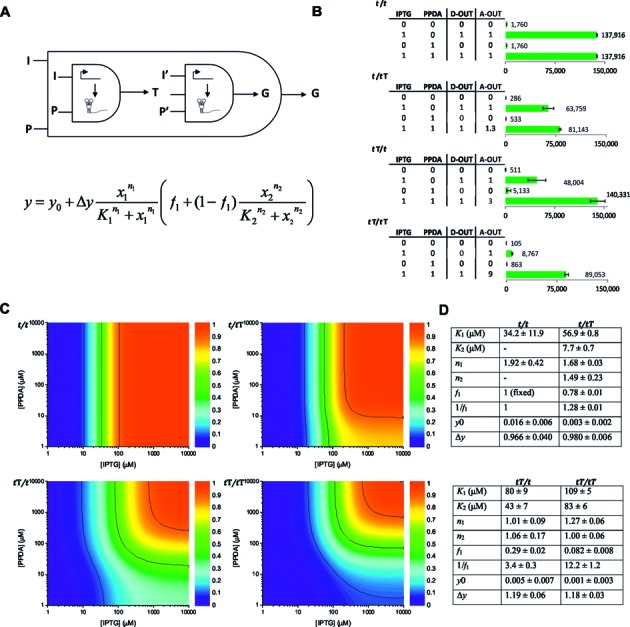
AND gate performance modelling. (**A**) Diagram and equation of the modular AND gate designed on the basis of the AND model presented in Kirouac *et al*. 2013, with a modification of the equation to allow for leaky expression in absence of PPDA (P), where *y*_0_ is the basal level of expression (RFU/OD) in the absence of both IPTG (I) and P, Δ*y* is the level of expression under saturation concentrations of both I (*x*_1_) and P (*x*_2_), *n* is the Hill coefficient and *K*_1_ and *K*_2_ are the EC_50_ values for I and P, respectively. The fraction of expression that occurs independent of P is determined by *f*_1_, such as when *f*_1_ = 1, expression is P-independent and when *f*_1_ = 0, the system behaves like a strict AND gate. The reciprocal, 1/*f*_1_, describes the enhancement in expression obtained under saturating I and P relative to that obtained with only I. (**B**) truth tables for the logic displayed by each expression system with an output defined with a 10% threshold cut off (D-OUT) and with an amplified output (A-OUT) showing the synergistic enhancement of using two inputs together over the sum of individual inputs [A-OUT = (I+P) / ((I)+(P))] with bar graphs of the measured output levels (RFU/OD). (**C**) Contour plots of the modelled I and P response for each expression system using the equation described in (**A**), black lines indicate the 10%, 50% and 90% contours. (**D**) Table of the global fitting parameters. The data represent the mean of at least two replicates with error bars showing standard errors.

The cascade control elements of the *t*T/*t*T system were categorized as two Boolean logic gates where gate one requires 2-inputs (IPTG and PPDA) to produce an output (T7 RNAP), and the second gate requires 3-inputs, both inducers (IPTG and PPDA) and T7 RNAP from the first gate, to produce the output (eGFP). For simplicity the system can be considered as operating overall with AND logic (Figure 2A). Truth tables were constructed to determine the operating function of the two inputs upon the various systems. Using a 10% threshold cut-off only the *t*T/*t*T system operated with AND Boolean logic, whereas the *t*T*/t* system was effectively an IPTG-only responsive gate (Figure 2B). However, this digital-type analysis belies the synergistic amplification (A-OUT) effect of using multiple layers of regulatory control. Here the performance output of *t*T/*t* with both inputs was greater (x3) than the sum of the individual induction inputs, and this synergistic amplification of input response was even greater (x9) when an additional ORS device was used in the *t*T/*t*T system.

A previously developed quantitative AND logic gate model (26) was modified to allow for ‘leaky’ expression, here permitting both single and dual-inputs to be assessed using a logistic Hill-type fitting parameter model (Figure 2A). Global EC_50_ values were determined for all systems (Figure 2C and D and Supplementary Figure S2). Higher EC_50_ values were observed upon the addition of regulatory control elements (*t*/*t* < *t*T/*t* < *t*T/*t*T), indicative of the additional regulatory checkpoints. The proximity of the EC_10_ and EC_90_ contours (Figure 2C) for *t*/*t* indicates a digital ON-OFF response of the transcriptional control, whereas the broader contour separation for *t*T/*t*T can be used to pre-select the optimal combination of inducers to achieve a desired level or titratable window of expression. The fraction of *eGFP* expression that occurs independently of PPDA (*f*_1_) for each system indicates that the model was consistent with the experimental data (Figure 2D). Indeed, the reciprocal (1/*f*_1_), which describes the enhancement in expression relative to IPTG induction alone, was 3-fold for the *t*T*/t* and 12-fold for the *t*T/*t*T systems. Further, the model indicates the copy number (*t*/*t* < *t*T/*t* < *t*T/*t*T), and positional (genome (*t*T/*t*) versus plasmid (*t*/*t*T)) effects of the ORS translational control device, where control of T7 RNAP is clearly the regulatory bottleneck within the synthetic cascade.

### Comparison against other tight expression systems

To more fully compare the expanded dynamic range and tight basal expression of the *t*T/*t*T system against other commonly used *E. coli* expression systems, we performed side by side analysis against T7 RNAP based systems that have been developed to reduce the basal leakage: BL21(DE3)pLysS (LysS), which constitutively expresses the T7 RNAP inhibitor T7 lysozyme (27), LEMO21(DE3) (Lemo) (9) based on the rhamnose inducible expression of a T7 lysozyme variant (LysY), the KRX strain based on the rhamnose inducible promoter (*rha*P_BAD_) (4,6). In addition we also compared the non-T7 RNAP pBAD system (pBAD), based on the arabinose inducible promoter (*ara*P_BAD_) (28,29). We initially compared the regulatory control (max/basal) and maximal output in small-scale cultures at short induction times (Supplementary Table S3). The *t*/*t* and KRX systems displayed the smallest window of regulatory control of 65- and 117-fold, respectively, Lemo, pBAD and LysS displayed modest dynamic ranges 315-, 387- and 459-fold, respectively, compared to the *t*T/*t*T system which displayed 845-fold. In terms of maximal expression under multiwell conditions, the systems based on the BL21(DE3) strain (*t*/*t*, LysS, Lemo) produced similar high expression levels (∼74–100%), with *t*T/*t*T and pBAD expressing ∼50% and ∼70%, respectively, and KRX produced the least (∼40%). In terms of relative basal control, *t*/*t* exhibited the greatest basal leak, followed by KRX and Lemo which exhibited only *ca*. 3-fold tighter basal control relative to *t*/*t*. LysS and pBAD displayed a *ca*. 6.5-fold reduction in basal, and *t*T/*t*T demonstrated the tightest control of all with *ca*. 20-fold tighter basal control relative to *t*/*t*.

The relative tunability of the various expression systems was assessed using bivariate matrix analysis (Supplementary Figure S3), and the dose-response data (Table 1 and Supplementary Table S4). Titration of the LysS system produced a DRLR of 5.8-fold indicative of small digital response window. Titration of the Lemo system with IPTG at fixed Rhamnose concentrations (100–1000 μM) produced DRLR responses of 27–35-fold indicative an intermediate digital-analogue response. Conversely, titration with Rhamnose at fixed IPTG concentrations (10–50 μM) produced DRLRs 11–14 indicating digital dose response curves. The KRX system, which in principle should be regulated by both Rhamnose and IPTG, displayed no significant change in expression upon IPTG titration (Supplementary Figure S3B), was however titrated by Rhamnose in an analogue dose response manner with a range of DRLRs 97–117 at different fixed IPTG concentrations. The pBAD system demonstrated a DRLR of 312 indicative an analogue dose response upon arabinose titration. The caveat to this observation is that arabinose uptake has been shown to demonstrate significant cellular-level heterogeneity previously (30), and also in this study (Figure 4B). Global performance modelling (Supplementary Figure S4) was generally consistent with the experimental data and demonstrated that KRX expression is dominantly controlled by Rhamnose induction, with IPTG resulting in only 1.15-fold (1/*f*_1_) effect upon expression above Rhamnose alone.

**Table 1.** Expression system nomenclature and performance summary

Strain	Code	Tuneability DRLR range^a^	%Max 5 h/20 h	Fold decrease of basal 5 h/20 h	Biomass	Cell viability^b^
BL21(DE3)_pET	*t*/*t*	10 (IPTG)	100/17	1/1	moderate	good
BL21(IL3)_pETORS	*t*T/*t*T	70–224 (PPDA) 18–22 (IPTG)	32/100	56/245	good	good
BL21(DE3)_pLysS_pET	LysS	5 (IPTG)	49/37	3/10	moderate	poor
LEMO21_pET	Lemo	11–14 (Rha) 27–35 (IPTG)	52/30	3/40	low	moderate
KRX_pET	KRX	97–117 (Rha)	17/49	18/66	low	moderate
BL21_pBAD	pBAD	312 (Ara)	36/39	34/224	good	good

Strain names, abbreviations and performance comparison for the novel expression systems reported in this study against of number of the commonly used *E. coli* expression strains and systems.

^a^Data extracted from the multiwell experiments performed at 30°C, 3 h. All other data extracted from UY shaker flask experiments performed at 30°C, 5 and 20 h.

^b^ Cell viability assessed by LDH activity harvested spent media.

### Volume and temperature scalability performance characteristics

The scalable performance of both *t*/*t* and *t*T/*t*T systems under batch high-cell density growth culture was performed in UY flasks at different induction temperatures. The *t/t* system performed best at 30°C after 5 h induction producing an eGFP volumetric yield of *ca*. 0.7 g/l, and a 28-fold induction of expression (Supplementary Table S5). This regulatory control was improved at lower temperature (265-fold) however at higher temperature a lower maximum yield was observed, along with a reduction in expression control (12-fold). The growth rate of the *t*/*t* system under induced conditions was also greatly affected at all temperatures above 20°C, where the doubling times were more than 2-fold slower than the un-induced cells (Supplementary Table S6). The *t*T/*t*T system (30°C, 5 h) showed lower volumetric yield (*ca*. 0.4 g/l), but maintained a large 423-fold induction of expression, and a 56-fold tighter basal control than for the *t/t* system, consistent with the multiwell-scale growth analysis (Table 1 and Supplementary Table S5). In addition, the growth rate of *t*T/*t*T system was also largely unaffected by induction, where the doubling times for both induced and un-induced cells were consistent at all temperatures (Supplementary Table S6).

We additionally compared the maximal output, regulatory control and basal leakage for the other *E. coli* expression systems in UY flasks (30°C, 5 h) (Supplementary Table S5). Maximal expression was observed for the *t/t* system, with the alternative strains expressing between 17–52% of this under full induction conditions (Table 1). The smallest regulatory control (max/basal) being the LysS, *t/t*, Lemo and KRX systems demonstrating between 28–84 fold control (Supplementary Table S5). The pBAD system demonstrated the next greatest dynamic range 345-fold control, with the *t*T/*t*T system demonstrating 500-fold control (Supplementary Table S5). In terms of relative basal control *t/t* exhibited the greatest basal leak, with LysS and Lemo exhibiting only 3-fold tighter basal control than *t/t*. The KRX system exhibited 18-fold reduction in basal leakage, whereas pBAD and *t*T/*t*T exhibited 34 and 56-fold tighter control respectively at 30°C, 5 h (Table 1).

### Kinetic performance characteristics

**Figure 3.**
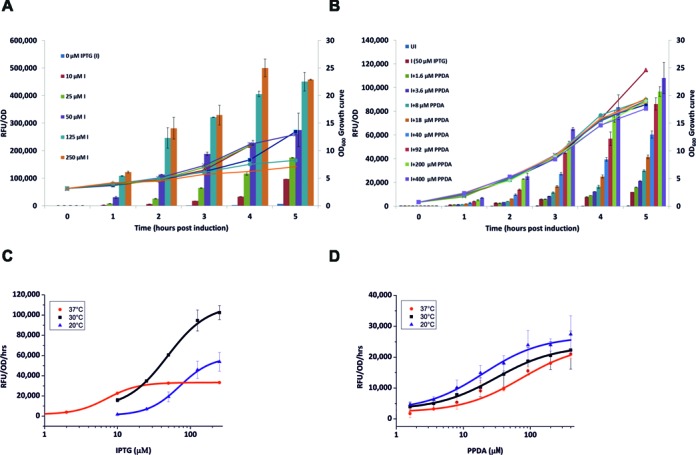
High cell density expression kinetics and performance. The *t*/*t* and *t*T/*t*T systems were grown at 30°C in 125 ml baffled flasks and induced when in log phase with the indicated concentrations (μM) of IPTG (I) and PPDA (P). Samples were collected at different times post induction and analysed for eGFP expression. **A-B**, normalized expression RFU/OD_600_ (bars) and absolute OD_600_ values (lines) were then plotted versus time for the *t*/*t* (**A**), and the *t*T/*t*T systems (**B**). (**C–D**) kinetic dose-response data fitted with a four-parameter logistic function of the RFU/OD values at different time points and induction temperatures for the *t*/*t* system titrated with IPTG (**C**), and the *t*T/*t*T system titrated with PPDA at a fixed IPTG concentration of 50 μM (**D**). The data represent the mean of at least two replicates with error bars showing standard errors.

To probe the effects of regulatory control upon cellular protein expression rates, a detailed kinetic analysis was performed with regular sampling of the expression cultures. Kinetic fitting (over 0–5 h) was used to calculate the specific rate (RFU/OD/h) and volumetric productivity (mg/l/h) of eGFP expression (Supplementary Figure S5 and Table S6). The *t/t* system, when fully induced, rapidly produced large amounts of protein at 30°C (*ca*. 102 × 10^3^ RFU/OD/h), this specific rate was highly dependent upon temperature, with these values dropping by 50% at 37°C. Indeed the ability to tune this specific rate expressed as DRLR was 30 at 30°C. Whereas, at lower and higher temperatures the specific rate response to IPTG operated with greater digital behaviour (DRLR *ca*. 10) (Figure 3 and Supplementary Table S7). The *t*T/*t*T system produced protein more slowly (*ca*. 23 × 10^3^ RFU/OD/h), and intriguingly was largely independent of temperature, indicating a robust balance between expression rate and biomass accumulation. Further the specific production rate could be precisely controlled, where the DRLR operated with consistent analogue behaviour (77–138) across all temperatures for both PPDA and IPTG titration. We further attempted to calculate the cellular expression rate for the pBAD system, upon induction this led to a modest initial burst (0–3 h), with max specific rate (*ca*. 40 × 10^3^ RFU/OD/h), after which expression reached a plateau (3–5 h) at all inducer concentrations (Supplementary Figure S5). The non-linear expression kinetics observed for pBAD is possibly related to the regulation of arabinose importer (araE) and cellular expression heterogeneity previously reported (30).

### Output continuance performance characteristics

At longer growth times (20 h) the *t/t* system only performed well at lower temperatures, with a basal expression leak of *ca*. 12% and volumetric yield of *ca*. 2.9 g/l at 20°C (Supplementary Table S5), indeed higher temperatures led to a dramatic decrease in output. The *t*T/*t*T system also showed a maximum volumetric yield of *ca*. 2 g/l at 20°C, and performed well at all temperatures tested with good basal control (0.2–0.5%), indicating the large window of induction is maintained at high cell density/longer expression times. Indeed at 30°C the basal control of the *t*T/*t*T system was >240-fold tighter than that of the *t/t* system (Table 1). The titration of the *t*T/*t*T system can also be observed visually by SDS-PAGE analysis of cell lysate, where eGFP expression equals *ca*. 30% of total cell protein (Supplementary Figure S6A). Comparison of protein solubility of the *t/t* and *t*T/*t*T systems at different expression times (Supplementary Figure S6B) also indicated that the majority of the recombinant protein is soluble, but that a greater proportion of insoluble eGFP is observed for the *t/t* system.

At extended growth periods (20 h) for all the *E. coli* expression systems induced at 30°C, maximal expression (RFU/OD) was observed for the *t*T/*t*T system with the other systems expressing between 17–49% of this under full induction conditions (Table 1). All systems based on the BL21(DE3) parental strain demonstrated poor or modest regulatory control (0–22-fold) due to basal leakage over the extended growth period, KRX displayed 55-fold, whereas pBAD and *t*T/*t*T demonstrated a regulatory control of 152- and 423-fold, respectively, indicating tight regulatory control at extended growth periods (Supplementary Table S5). In terms of relative basal control the *t/t* system exhibited the greatest basal leak, with LysS exhibiting only a 10-fold tighter basal control than *t/t*. Lemo and KRX exhibited 40 and 66-fold reduction in basal leakage, and pBAD and *t*T/*t*T exhibited 224- and 245-fold tighter control respectively (Table 1). To probe for cell viability at these extended growth periods we performed a membrane leakage analysis, using a lactate dehydrogenase activity (LDH) assay on *eGFP* expression systems (Supplementary Figure S7). Values were expressed as a percentage LDH activity observed from the media relative to chemically lysed cells. Under un-induced conditions KRX and Lemo exhibited the highest LDH activity (8–15%) indicating a modest compromise in cell viability, the remaining strains demonstrated basal LDH activities of 3–5%. Under induced conditions the majority of the T7 RNAP based expression systems demonstrated a 1.4–2.6-fold increase in LDH activity, with the *t*T/*t*T exhibiting the lowest overall activity at 7.7%, with Lemo and KRX exhibiting 12.6 and 21.0%, respectively. These cell viability measurements are consistent with the observed biomass accumulation where Lemo and KRX biomass is 1.6- and 2.3-fold lower than *t*T/*t*T (Supplementary Table S5). The LysS system exhibited significant LDH activity levels of 47.2% consistent with the previously reported amidase activity of T7 lysozyme, constitutively expressed from pLysS (31). The lowest activity was demonstrated by the pBAD system with LDH activity of 3.5%.

### Single-cell expression analysis

**Figure 4.**
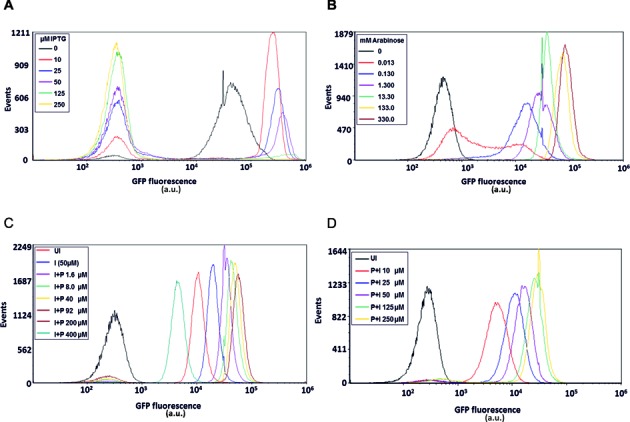
Single-cell expression analysis. (**A–D**) flow cytometry analysis of *t*/*t* versus IPTG (**A**), BL21 pBAD-eGFP versus arabinose (**B**), *t*T/*t*T versus PPDA with fixed 50 μM IPTG (**C**) and *t*T/*t*T versus IPTG with fixed 92 μM PPDA (**D**). Cell populations were grown at 30°C for 20 h under different conditions, (uninduced (UI), IPTG (I) 0–250 μM, PPDA (P) 0–400 μM, arabinose (Ara) 0–330 μM). The data show that the *t*T/*t*T system possesses greater cellular homogeneity and stability in gene expression.

In agreement with the population level data (Supplementary Table S1), flow cytometry analysis of the *t/t* system 20 h post-induction confirmed the high basal expression in the absence of inducer, presumably operating via an auto-induction mechanism (Figure 4A) (32). Increased IPTG concentration was associated with greater heterogeneity, expressed as coefficients of variation CV (Supplementary Table S8A). This increased variation, was due to the occurrence of a low intensity fluorescent sub-population of cells, which dominated at higher IPTG concentrations (≥125 μM), this observation has previously been reported (33). This effect was not observed after a shorter induction time (5 h) (Supplementary Figure S8).

By contrast the *t*T/*t*T system did not exhibit a significant low intensity fluorescent subpopulation (Figure 4C,D). In all cases at least 89% of the total population expressed eGFP within a favourable homogenous region as indicated by low CV values (Supplementary Table S8C and D). The data support the population level observations that titratable control was afforded with both IPTG (Figure 4C) and PPDA (Figure 4D). Tight control over basal expression was evidenced by a lack of leakiness within un-induced cells, where the mean eGFP expression of the total population remained low.

As a comparison, eGFP expression controlled by the arabinose inducible (pBAD) system was also analysed by flow cytometry (Figure 4B). This system demonstrated tight basal control, titratable induction of expression and high maximal expression intensity. However, consistent with previously reported findings (30), at sub-maximal inducer concentrations (0.013 mM) variation in eGFP expression was substantial (Supplementary Table S8B).

### Exemplar proteins

**Figure 5.**
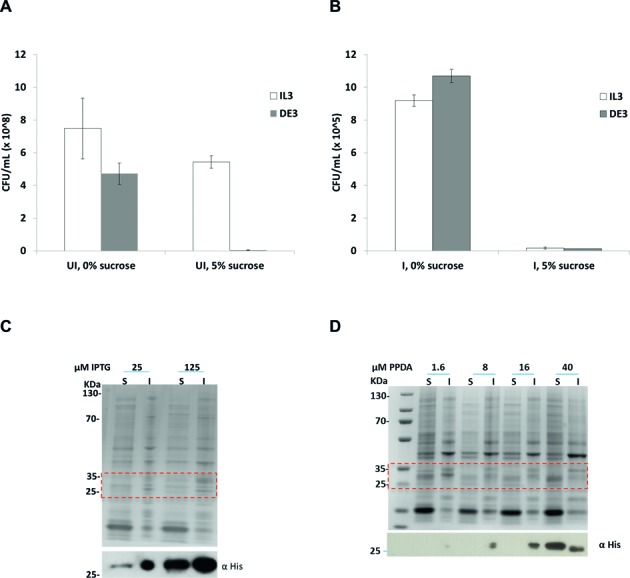
Expression of toxic and challenging proteins. (**A–B**) Comparison of colony-forming unity (CFU) from *sacB* assay, CFU/ml counts for both *t*/*t* and *t*T/*t* systems carrying a pET15-sacB plasmid were determined in the absence (**A**) and presence (**B**) of inducers with and without the addition of 5% sucrose. In the absence of inducers (UI), expression of the *sacB* gene led to highly sucrose-sensitive behaviour in the *t*/*t* system, whereas upon induction (I/P, 50 μM IPTG plus 100 μM PPDA) both systems displayed sensitivity. The data represent the mean of at least three replicates with error bars showing standard deviation errors. (**C–D**) solubility analysis of the scFv protein, visualized by SDS-PAGE and western blot, of soluble (S) and insoluble (I) fractions. *t*/*t* was induced for 3 h at 30°C (**C**) and *t*T/*t*T for 20 h at 30°C (**D**), with the indicated concentration of inducer.

We sought to demonstrate the performance of the novel systems for the expression of both toxic and clinically relevant proteins. We validated the tight basal control observed for the *RiboTite* system by controlling the expression of the toxic gene encoding levansucrase (*sacB*) (34–36). In the presence of 5% sucrose, the uninduced *t*T/*t* system maintained good cell viability, indicating tight control over basal expression, whereas *t*/*t* system's cell viability was comprised, indicative of leaky basal expression (Figure 5A). Upon induction in the absence of sucrose, we observed a 2-order of magnitude decrease in cell viability for both strains, presumably due to the burden of recombinant protein expression and/or plasmid loss (Figure 5B). Induction in the presence of sucrose led to a pronounced loss of viability for both strains, consistent with the efficient expression of *sacB*. The titratability and tight basal control for the *t*T/*t*T system was further demonstrated for a range of clinically relevant proteins, such as the metabolite cancer target MTH1 (37,38), the cytokine Interferon α2a, and the tumour suppressor protein p53 by western blot analysis (Supplementary Figure S9). For all target proteins both tight basal control and titration expression was observed for the *t*T/*t*T system.

In order to assess whether the tight basal expression, slow specific expression rate, and/or the tuneable homogenous expression of the *t*T/*t*T system could offer some further advantage, we sought to explore its use for the expression of a protein prone to insolubility and a toxic peptide. The IgG antibody fragment scFv163R4 is produced as soluble and insoluble forms when expressed in the cytoplasm of *E. coli* (39). To challenge the *t*T/*t*T system we looked to utilize the expanded tuneable expression range, and the slower specific productivity of the system to influence the amount of soluble scFv produced. Initially we compared the titration window for both *t*/*t* and *t*T/*t*T systems (Supplementary Figure S9B, 9C). A large window of regulation was observed for the *t*T/*t*T system upon titration of PPDA, whereas a narrow window of regulation upon IPTG titration was observed for *t*/*t*, consistent with the eGFP expression. At low IPTG concentration the *t*/*t* system produced scFv mostly in the insoluble fraction, contrary to the expected, at higher IPTG concentration the system produced roughly equal soluble and insoluble fractions (Figure 5C). Both Lemo and LysS systems similarly produced the scFv in both roughly equal soluble and insoluble forms (Supplementary Figure S9D). Intriguingly induction of the *t*T/*t*T system with low PPDA concentrations produced mainly the in-soluble form of scFv, however at the optimal PPDA inducer concentration (40 μM), scFv was dominantly produced in soluble form (Figure 5D).

We also expressed the type II-like bacteriocin, epidermicin NI01 from *S. epidermidis* (22), previously expressed only at 10 μg/l (personal communication, M. Upton). Epidermicin has potent activity against Gram-positive bacteria, but not against *E. coli*. However, accumulation in the cytoplasm of *E. coli* is toxic and significantly limits yields. The expression systems containing the plasmid encoding the toxic peptide were induced at different inducer concentrations (Supplementary Figure S10). Titration of pBAD and *t*T/*t*T is observed; this is further confirmed for the *t*T/*t*T for western blot analysis (Supplementary Figure S10B). In addition we sought to qualitatively compare the production capabilities of three expression systems (Supplementary Figure S10C). The specific expression yields (target/total) were obtained by image densitometry analysis, where it can be seen that *t*T/*t*T expressed epidermicin ∼13%, pBAD at ∼12%, and *t*/*t* expression at ∼6%. Finally cell growth in rich media of the expression systems was monitored (Supplementary Figure S10D, E). In the absence of induction, both pBAD and *t*T/*t*T reached high cell density OD_600_ ∼17, and *t*/*t* demonstrated slightly compromised biomass of OD_600_ ∼15. As toxic protein expression can result in arrest of cell growth upon induction, an optimal mid-phase induction of OD_600_ = 8 was selected. Induction led to a rapid halt in cell growth for all systems, leading to a plateau in biomass accumulation (5–10 h), and then a slow increase growth for the *t*/*t* and pBAD systems resulting in final OD_600_ ∼10 (23 h), a 30–43% reduction in biomass compared to the un-induced conditions. Whereas the *t*T/*t*T system after the plateau in growth, recovered and continued to grow reaching a final OD_600_ = 15, a biomass reduction of only *ca*. 9%, relative to uninduced, and 42–52% greater biomass than the *t*/*t* and pBAD systems.

## DISCUSSION

The *t*/*t* system, operating with dual transcriptional control only, demonstrated leaky basal expression and a narrow dynamic range of ligand response, indicating a digital (ON/OFF) dose response to IPTG induction. The system was also sensitive to growth temperature, where opposing affects were observed. Increasing temperature led initially to a 2-fold increase in both specific yield (RFU/OD), and specific rate (RFU/OD/h), however further temperature elevation to 37°C led to 2-fold reduction in these performance parameters. The fragility of the system was also demonstrated by the effect of induction upon doubling times (Supplementary Table S6). Furthermore, for extended growth times under batch (UY) conditions the system performed poorly at temperatures above 20°C, with loss of IPTG dependent regulatory control, population heterogeneity and reduced yield. Various attempts have been made to alleviate these limitations (1,9), and both the digital response and stoichiometry of the *lac* repressor-IPTG interaction have been widely studied (40,41).

The *t*T/*t* system demonstrated robust regulatory control, exhibiting >400-fold induction after 3 h at 30°C (multiwell scale), which was reasonably maintained at extended growth times (56-fold after 20 h), conditions under which the *t/t* system displayed only 65-fold and 3-fold control, respectively. The system also displayed a comparable maximal expression output relative to the *t/t* system. Finally, a DRLR of 79 for the *t*T/*t* system (in response to PPDA), compared to a DRLR of 10 for the *t*/*t* system (in response to IPTG), indicates a highly titratable analogue dose response, demonstrating the enhanced potential of dual transcriptional and translational control over T7 RNAP.

The *t*T/*t*T system at 30°C (multiwell scale), afforded a large induction control (*ca*. 850-fold), the lowest basal expression levels (0.1%), and a significantly expanded DRLR of 245, allowing for simple and precise analogue titration of target gene expression levels. The IPTG response behaviour at fixed PPDA concentrations indicates the system functions like a rheostat, allowing regulation of the maximal output (vertical extension) without affecting basal output or other expression parameters. Alternatively, varying IPTG could attenuate both the PPDA analogue response behaviour and max/basal expression levels. Where at low (IPTG) transcriptional levels the PPDA sensing window (DRLR) is greatly extended, producing horizontal scaling of the response curves, and at high transcript levels an increase in both max/basal expression is observed producing a vertical scaling of expression response. This demonstrates that the performance and response of the system can be precisely tuned to permit dynamic control upon addition of small molecule inducers. At larger scale (UY shaker flasks), the *t*T/*t*T maintained its tight basal control at both short and extended growth periods, demonstrating >240-fold (20 h) tighter basal than *t*/*t*.

The *t*T/*t*T system performed well against all the commonly used *E. coli* expression systems used in this benchmarking study. Across all scales and conditions the basal control was the most tightly controlled and tunability was best maintained for the *t*T/*t*T system. The Lys system reduced basal expression of 3–10-fold relative to *t*/*t*, but also resulted in a reduced maximal expression (UY scale) and displayed a compromised viability, most likely due to amidase activity of T7 lysozyme (31). Lemo operated with intermediate-digital titratable control, a modest reduction in basal expression, moderate expression levels (UY scale), but moderately poor cell viability and low biomass. Similarly KRX operated with good analogue control, modest reduction in basal, moderate expression levels, but moderately poor viability and poor biomass under the conditions assessed. The pBAD system operated with excellent analogue control (DRLR = 230), impressive basal control, and good viability and biomass, however was subject to expression heterogeneity at sub-maximal inducer concentrations, consistent with previous reports (3,30). T7 RNAP-based expression systems are known to suffer from growth defects and compromised cell viability, likely due to the speed of T7 RNAP, which leads to the desynchronisation of transcription-translation (42). Of all the T7-based systems tested *t*T/*t*T was able to grow to the highest cell density and exhibited the least compromise to cell viability under induced conditions. In terms of maximal protein expression from shaker flasks at 30°C the *t*T/*t*T system was only out-performed by the BL21(DE3) based systems at short induction times (5 h), at longer induction times the *t*T/*t*T system produced the highest specific and volumetric expression levels.

Kinetic protein expression analysis (UY scale) demonstrated that the *t*T/*t*T system operates with a lower maximal specific rate compared to the benchmark *t*/*t* system, and that this rate demonstrated little dependency upon temperature. Intriguingly, the specific production rate could be tunably controlled, with consistent analogue behaviour, by both PPDA and IPTG over the range of temperatures assessed. Both tuneable control and tight basal control of expression was maintained for extended durations far after media glucose consumption, conditions where stand-alone *lac* operator/repressor systems are known to suffer from extensive leak via catabolite de-repression (32). In addition, the system maintained tuneable expression within a tight homogenous range across the whole cell population in response to both inducers. It has been shown previously that lowering the expression temperature <25°C can enhance protein yield (43,44), and the accepted wisdom is that lower temperature <20°C can enhance protein solubility and yield. However at temperatures ≤20°C *E. coli* growth rapidly drops, and at lower temperatures the cellular processing capacity, e.g. chaperone activity is compromised (45). Attempts to address this conflict have indeed been sought by co-expression of chaperones from psychrophilic bacterium (46). However, the ability to slow and regulate expression rate, as demonstrated with the *t*T/*t*T system, without compromising cellular (enzymatic) processing capacity, due to a drop temperature would be advantageous. Additionally on a technical level for the production of recombinant proteins at a pilot or industrial scale, cooling of large bioreactors can be a costly and technical challenge.

For the expression applications with clinically relevant and/or toxic proteins of interest, the *t*T/*t*T system performed well, enhancing the fraction of soluble scFv produced relative to the t/t system. Further the *t*T/*t*T system demonstrated the enhanced volumetric expression of the antimicrobial peptide epidermicin NI01 2-fold more than *t*/*t*.

In summary, the dual transcriptional-translational regulatory cascade afforded by the *t*T/*t*T expression system, comprising a dual *lac*-operator/repressor and orthogonal riboswitch controlling both T7 RNAP and the gene of interest, offers the greatest regulatory performance of all the expression systems assessed in this study. This novel expression system provides: (i) tight control over leaky basal expression in the absence of small molecule induction, permitting up to 850-fold induction; (ii) access to high levels of induction with little or no comprise to cell growth or viability; (iii) enhanced cellular level expression homogeneity; (iv) predictable specific productivity rates across a range of temperatures; and (v) an expanded dynamic range of ligand response, greatly improving the titratability of target gene expression. The PPDA inducer is readily prepared from cheap starting materials (47), and its costs is not likely to hinder use for large-scale expression. Other efforts to reduce the activity and regulate T7 RNAP have included use of allosteric inhibition (8), more tightly controlled inducible promoters (1), stress engineered mutant strains (7), repressible allosteric regulation (9), T7 RNAP fragmentation (18,19) and T7 promoter engineering (31). However, the approach described here is the first report of the highly active T7 RNA polymerase being controlled at both the transcriptional and translation level. This inducible synthetic system emulates nature by using multiple points of regulatory control, including additional post-transcriptional control mechanisms, permitting a robust balance between expression rate (ribosome capacity) and biomass accumulation (cellular resource) (4,6,27).

We postulate that the lower specific productivity rate of the *RiboTite* system, due to the translational ON control afforded by the use of orthogonal riboswitches, might be useful for a range of protein production, metabolic engineering and synthetic biology applications. By matching the rate of protein expression to the downstream cellular processing capacity, cellular viability could be maintained, whilst maximizing both specific productivity and yield. In the case of more complex metabolic engineering, this characteristic would permit flux optimization by fine-tuning expression of a specific gene within an engineered biosynthetic pathway.

## Supplementary Material

SUPPLEMENTARY DATA
