# Supplementary material for: Dual transcriptional-translational cascade permits cellular level tuneable expression control
Source: Nucleic Acids Res. 2015 Sep 23;44(3):e21. doi: 10.1093/nar/gkv912 (PMC4756846; doi:10.1093/nar/gkv912)
Supplement: SUPPLEMENTARY DATA [file supp_44_3_e21__index.html]

Dual transcriptional-translational cascade permits cellular level tuneable expression control — SUPPLEMENTARY DATA 

# Dual transcriptional-translational cascade permits cellular level tuneable expression control

## SUPPLEMENTARY DATA

- SUPPLEMENTARY DATA
