# Supplementary material for: Dual transcriptional-translational cascade permits cellular level tuneable expression control
Source: Nucleic Acids Res. 2015 Sep 23;44(3):e21. doi: 10.1093/nar/gkv912 (PMC4756846; doi:10.1093/nar/gkv912)
Supplement: SUPPLEMENTARY DATA [file supp_gkv912_nar-01659-met-g-2015-File008.pdf]

SUPPLEMENTARY DATA:

Dual transcriptional-translational cascade permits cellular level tuneable expression control

I. Supplementary Figures and Tables Legends……………...........…………………….2

II. Supplementary Figures 1-10………………………………………………………4

III. Supplementary Tables 1-8……………………………………………...………….5

Supplementary Figures and Tables

Supplementary Figure S1. Bivariate multi-well expression analysis The different systems were grown at 30°C in deep 96-well plates and induced when in log phase with various combinations of IPTG and PPDA to create a bivariate matrix. Samples were collected and analyzed for eGFP relative fluorescent units (RFU) at: 3hrs growth post induction for *t*/*t*T system **(A),** 20hrs growth post induction for *t*/*t* (**B**), *t*/*t*T (**C**), *t*T/*t* (**D**), and *t*T/*t*T (**F**) systems. Normalized RFU against OD_{600} were then plotted in the matrix format showed. The data represent the mean of at least two biological replicates.

Supplementary Figure S2. Global fitting of the combined IPTG and PPDA-dependent expression response for the different systems analyzed The data collected for the experiment described in Figure 1 (B-I) were globally fitted with the equation showed in Fig 2a and the resulting modelled behavior and parameters in Fig 2b**.** Global normalized RFU against OD_{600} were then plotted in the global matrix format showed. The error bars show standard error of at least 2 biological duplicates.

Supplementary Figure S3. Bivariate multi-well expression analysis of current *E. coli* expression systems developed for tight basal control. eGFP expression matrices and dose response curves, for relative fluorescent units (RFU) normalized to cell density (OD_{600}) under different inducer concentrations at 30°C, 3hrs post induction for the systems: pLysS (**A**), KRX (**B**) pBAD (**C**) and Lemo (**D**). Dose response curves were fitted to a four-parameter logistic function. The data represent the mean of at least two biological replicates with error bars showing standard error.

Supplementary Figure S4. Global fitting of parameters of expression performance for different E. coli strains. Global normalized RFU against OD_{600} were plotted in the global matrix format showed (**A-B**). The error bars show standard errors of at least 2 biological duplicates. Contour plots of the modelled inducer response for each expression system using the equation described in Fig. 2A, black lines indicate the 10%, 50% and 90% contours (**C-D**). KRX (**A, C**), LEMO (**B, D**) (Note that the axes are reversed in the KRX plot).

Supplementary Figure S5. Expression kinetic fitting data (RFU/OD *vs*. Time). The *t*/*t* and *t*T/*t*T systems were grown at 30°C in 125 mL baffled flasks whereas the pBAD system at 37°C; all the system where induced when in log phase with the indicated combination of inducers. Samples were collected at different post induction times and analyzed for eGFP RFU. Normalized RFU against OD_{600} were then plotted versus post induction time to detect the linear range of dose-response curve for the *t*/*t* (**A**), the *t*T/*t*T [(**B**, PPDA) (**C**, IPTG)] and pBAD (**D**) systems. The data represent the mean of at least two biological replicates with error bars showing standard error.

Supplementary Figure S6. eGFP PPDA modulation production and solubility by *t*T/*t*T system. The *t*T/*t*T system was grown at 30°C in 125 mL baffled flasks and induced when in log phase with 50 μM of IPTG and various concentration of PPDA (1.6, 8, 16, 40 and 200 μM). Samples were collected after 20hrs of induction and the same equivalent of OD_{600} units were loaded on a SDS 4-20% polyacrylamide gel (**A**). Soluble and insoluble fractions were separated and compared against similar fractions from the *t*/*t* system

(3hrs induction) by SDS 4-20% polyacrylamide gel analysis (**B**). The % values indicate the abundance of the protein of interest relative to total cell proteins and were calculated by densitometry analysis.

Supplementary Figure S7. Cell viability LDH assay for all *E. coli* expression systems under both fully induced and un-induced conditions 20hrs at 30°C. The data represent the mean of at least two biological replicates with error bars showing standard error.

Supplementary Figure S8. Population level analysis. Flow cytometry analysis of *t*/*t* vs. IPTG. Cell cultures were grown at 30°C for 5 hrs under different conditions (uninduced (UI), IPTG 0-250 μM).

Supplementary Figure S9. Western blot analysis of clinically relevant proteins The *t*T/*t*T system carrying the indicated gene target was grown at 37°C in baffled flasks and induced in log-phase with the indicated combination of IPTG and PPDA for 4 hours. The samples were then collected and similar amount analyzed for the expression by western blot visualized with α His antibody: MTH1**,** IFNa2a**,** p53 **(A)**. Expression of the scFv gene from the *t*T/*t*T (50 μM IPTG, PPDA 1.6-200 μM, 20hrs, 30°C) (**B**), *t*/*t* **(**250 μM IPTG, 3hrs, 30°C) (**C**), the Lemo and LysS systems (under the inducer conditions indicated for 3hrs, 30°C) (**D**). α RNA pol σ^{70} was used as loading control.

Supplementary Figure S10. SDS-PAGE and western blot analysis of toxic peptide expression The toxic peptide Epidermicin NI01 was expressed from different expression systems after 3hr induction at 37°C. Titration was observed via SDS-PAGE by stain-free in-gel fluorescence for the tT/tT (IPTG 100μM, PPDA 0-400μM) and pBAD (arabinose 0-330 μM) systems (**A**). Expression and titration of Epidermicin NI01 from the tT/tT (IPTG 100μM, PPDA 0-400μM) system visualized by western blot (**B**). A qualitative comparison of expression was performed by SDS-PAGE analysis of the tT/tT, pBAD, and t/t systems (band intensity as a percentage lane intensity shown) (**C**). Growth curves for the pBAD, t/t and tT/tT systems under uniduced (**D**) and induced conditions (arabinose 133μM; IPTG 100μM; IPTG 100μM + PPDA 200μM) (**E**). PC: Purified Control (NI01).

Supplementary Table S1. Bivariate multi-well expression maximum and minimum data RFU/OD for the indicated systems at 30°C, 3 and 20hrs post induction. The data represent the mean of at least two biological replicates with standard error.

Supplementary Table S2. Dose-response curves fitted with four-parameter logistic function of the multi-well expression data (RFU/OD) at 30°C, 3 hrs post induction for *t*/*t* (a), *t*/*t*T (b), *t*T/T(c) and *t*T/*t*T (d) system. The equation used for the fitting is: $y = A2 + (A1-A2)/(1 + (x/x_0)^n_H)$ where A1 and A2 are the min and max RFU/OD value respectively, x_0 is the center of the curve (EC_{50}) and n_H is the Hill coefficient.

Supplementary Table S3. Bivariate multi-well expression maximum and minimum data RFU/OD for the indicated systems at 30°C, 3hrs post induction. The data represent the mean of at least two biological replicates with standard error.

Supplementary Table S4. Dose-response curves fitted with four-parameter logistic function of the multi-well expression data (RFU/OD) at 30°C, 3 hrs post induction for LysS, LEMO, KRX, and pBAD systems. The equation used for the fitting is: $y = A2 + (A1-A2)/(1 + (x/x_0)^\wedge n_H)$ where A1 and A2 are the min and max RFU/OD value respectively, x_0 is the center of the curve (EC_{50}) and n_H is the Hill coefficient. Rha is for rhamnose and Ara is for arabinose.

Supplementary Table S5. Comparison of eGFP production at maximal level between the indicated systems in UY flask growth at indicated temperature and time. *t*/*t* and LysS systems induced with 250 µM IPTG, *t*T/*t*T system with 200 µM IPTG and 1mM PPDA, Lemo system with 250 µM IPTG and 0.1mM Rha, pBAD system with 5% Ara, KRX system with 250 µM IPTG and 2mM Rha. The data represent the mean of at least two biological replicates with standard errors.

Supplementary Table S6. Comparison of eGFP production at maximal levels, doubling times and specific rates between the *t*/*t* and *t*T/*t*T systems in UY flask growth at indicated temperature at 5hrs. The data represent the mean of at least two biological replicates with standard errors.

Supplementary Table S7. Dose-response curves fitted with four-parameter logistic function for UY flask specific expression rate data (RFU/OD/hr) at different temperatures for the indicated systems. The equation used for the fitting is as described in Table S2.

Supplementary Table S8. FACS data for all and gated populations under different induction conditions for the following systems grown for 20hrs after induction at 30°C: *t*/*t* vs IPTG (a), *t*T/*t*T system vs PPDA at fixed IPTG (50 µM) (c) and vs IPTG at fixed PPDA (92 µM) (d) and pBAD system vs arabinose (b) grown for 20hrs after induction at 37°C.

Supplementary Figure 1

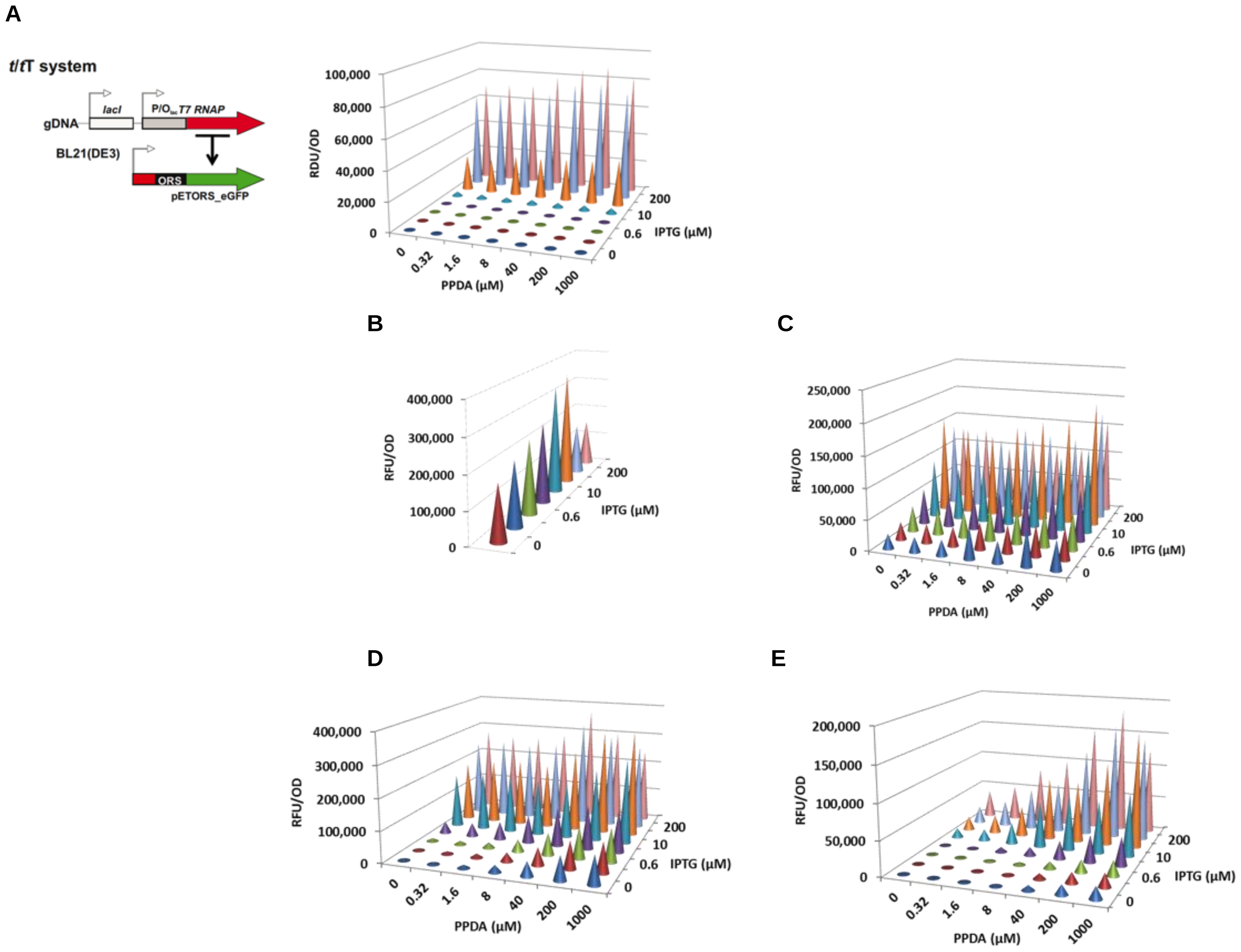

Supplementary Figure 2

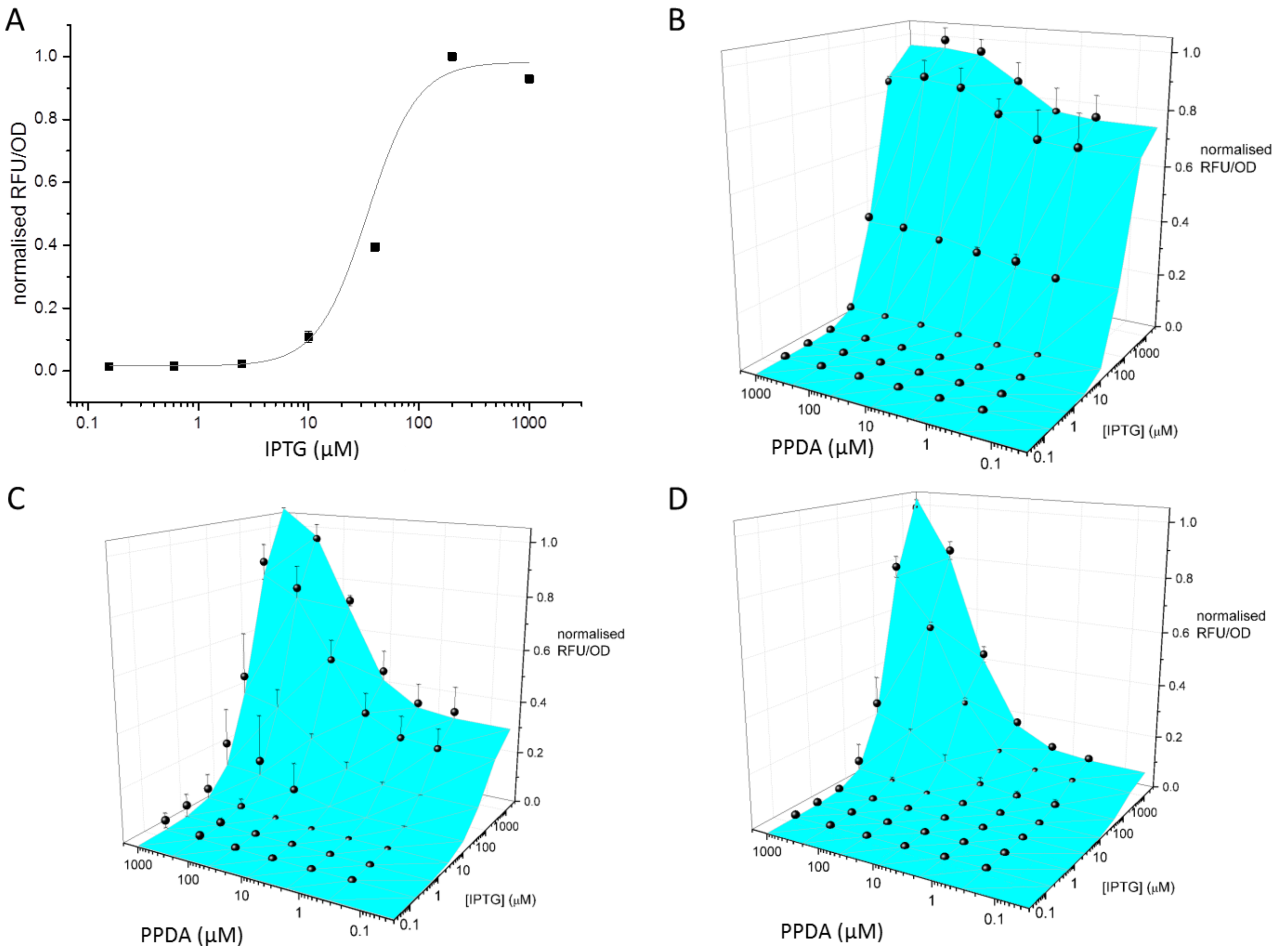

Supplementary Figure 3

A B C D

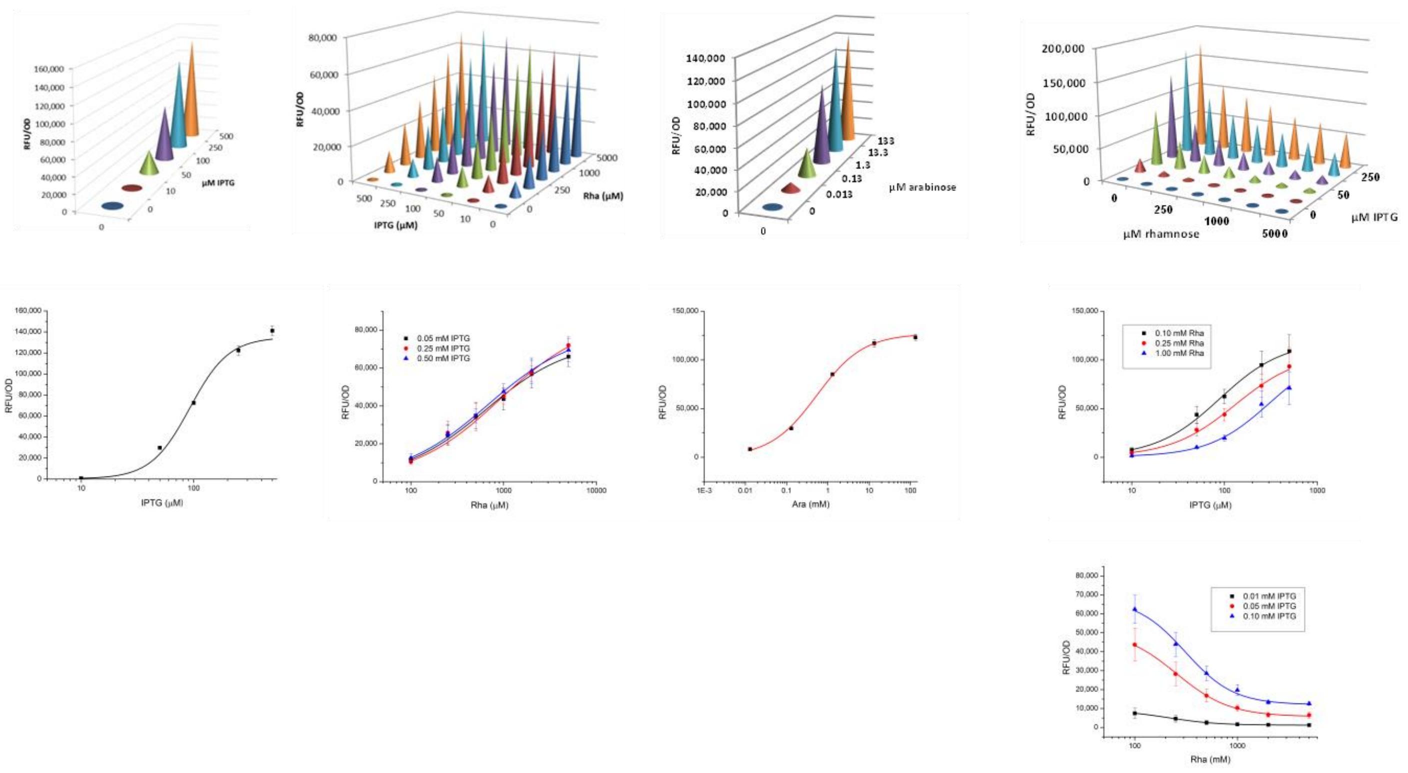

Supplementary Figure 4

A

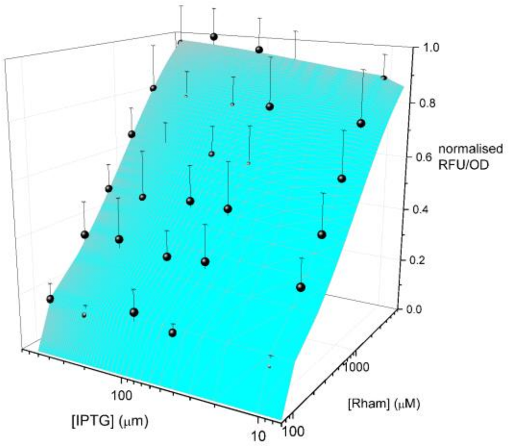

B

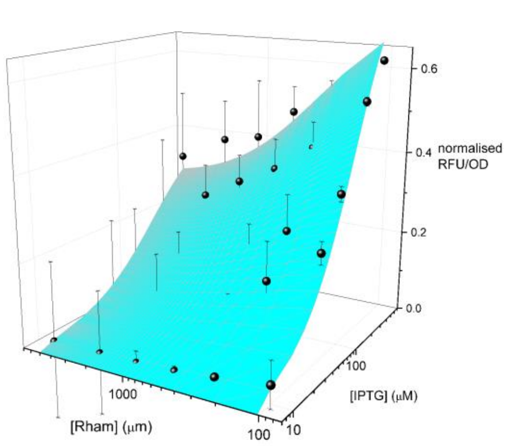

C

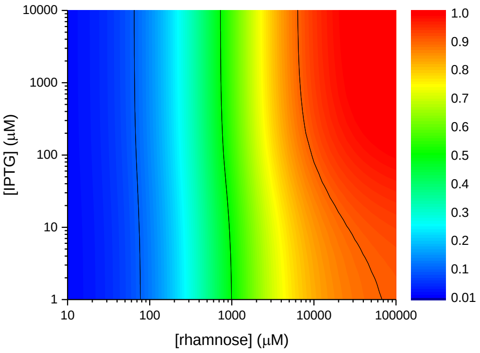

D

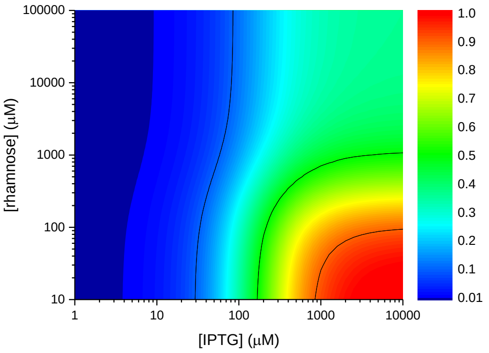

E

	experiment	
	KRX[a]	LEMO
K_1 (μM)	791 ± 62	174 ± 334
K_2 (μM)	42 ± 43	311 ± 861
n_1	0.86 ± 0.04	1.2 ± 1.3
n_2	0.8 ± 0.5	-1.1 ± 3.0
f_1	0.87 ± 0.04	0.35 ± 0.63
$1/f_1$	1.15 ± 0.05	2.9 ± 5.1
$y0$	0.010 ± 0.008	0 (fixed)
Δy	1.199 ± 0.057	1 (fixed)

a. Fit with x_1 = rhamnose and x_2 = IPTG as these experiments are essentially independence of [IPTG]. b. $n_2 < 0$ as RFU decreases with increasing rhamnose (x_2). This strain has a fairly strong digital response at low [rhamnose].

Supplementary Figure 5

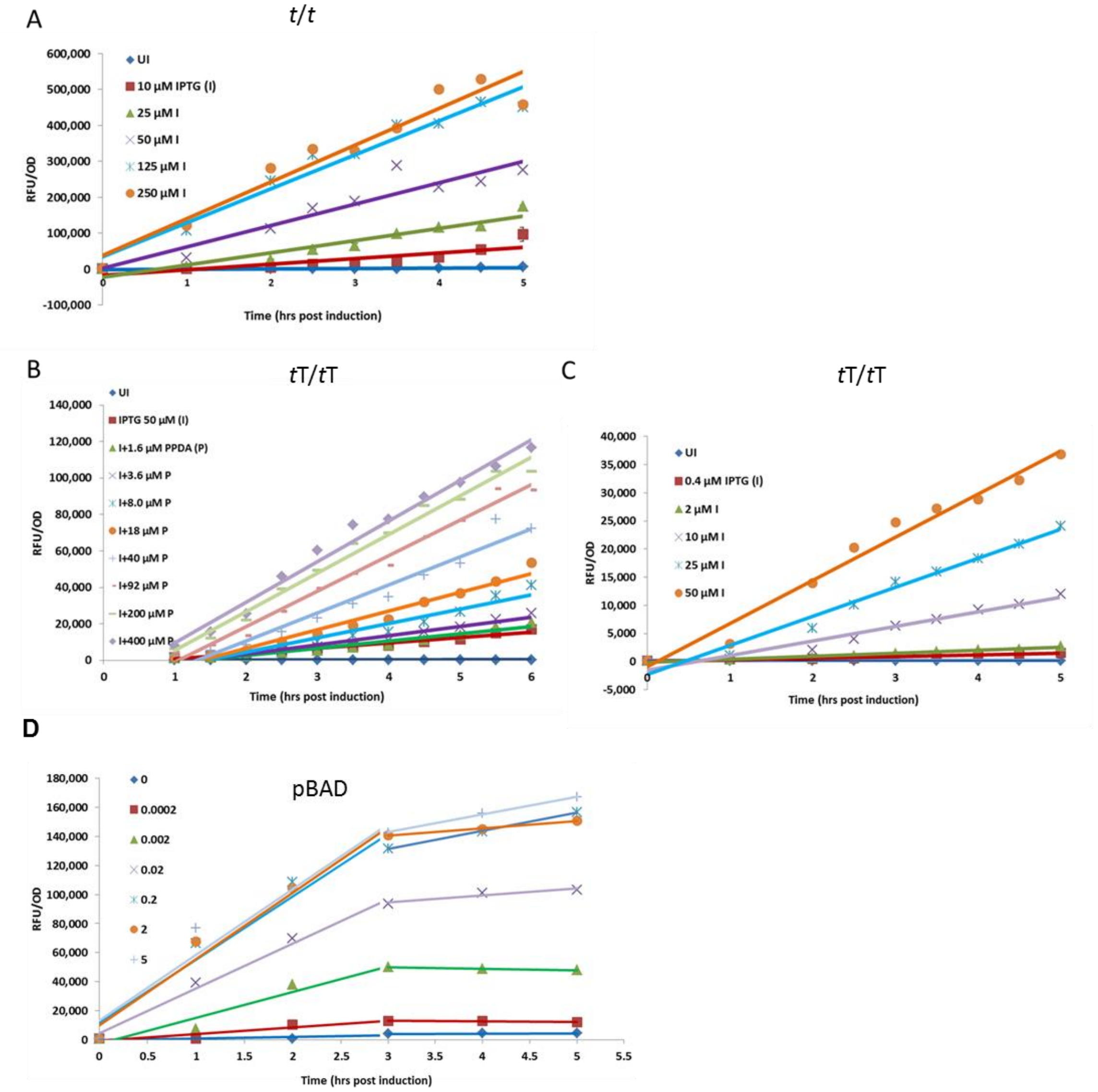

Supplementary Figure 6

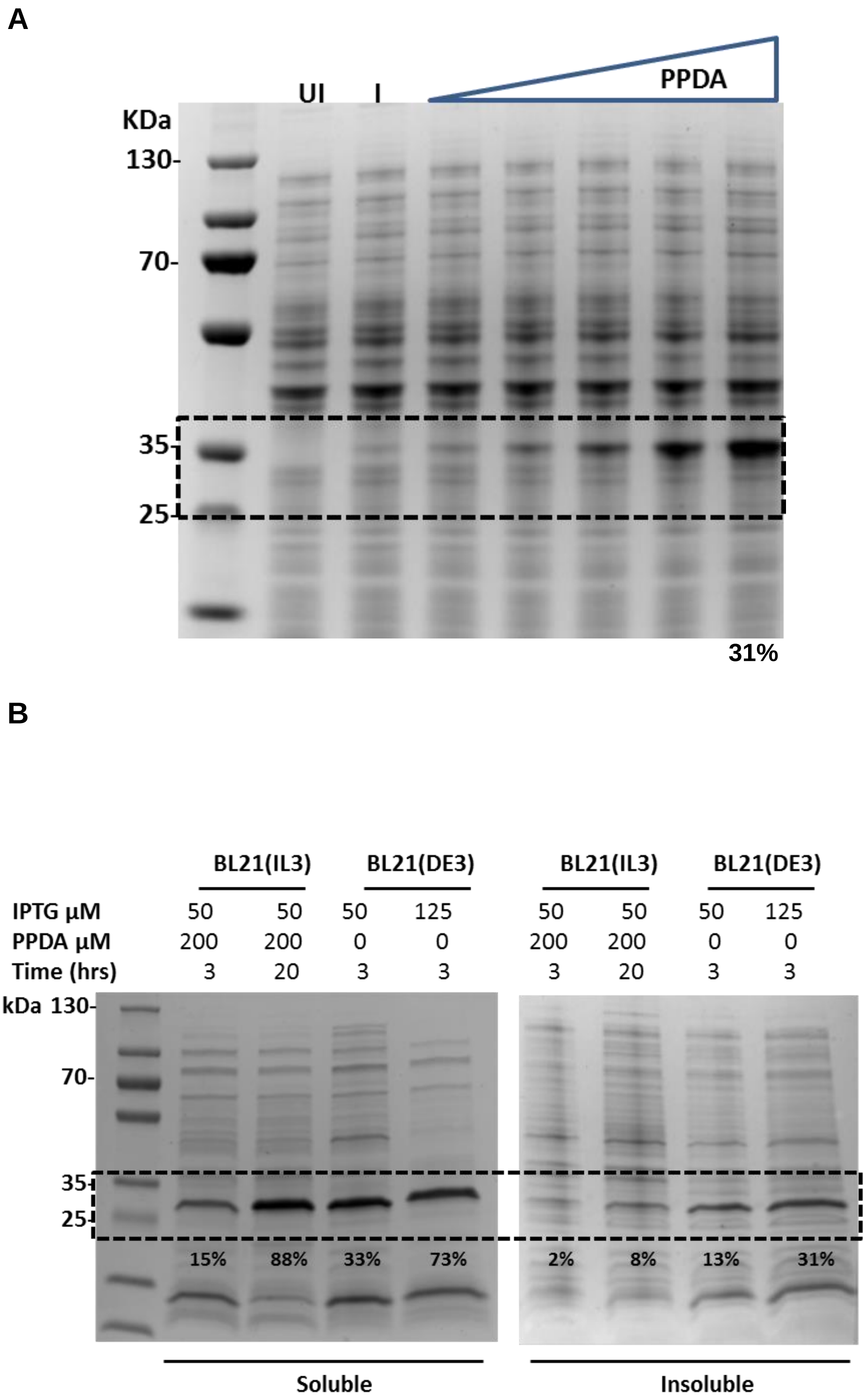

Supplementary Figure 7

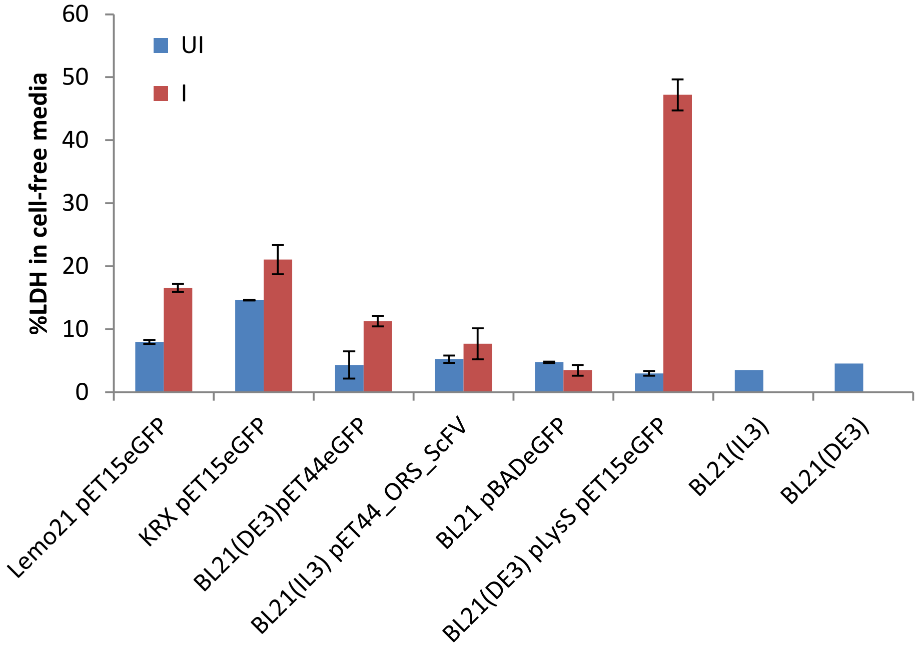

Supplementary Figure 8

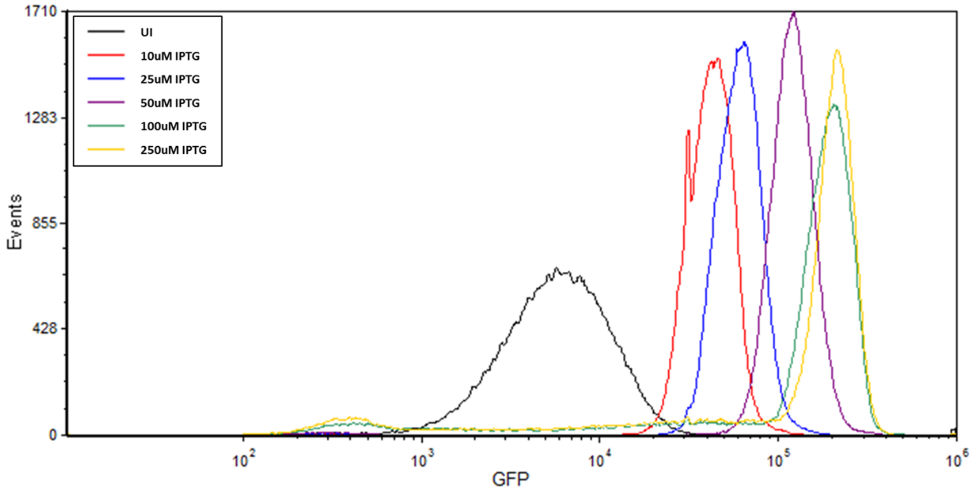

eGFP fluorescence (a.u.)

Supplementary Figure 9

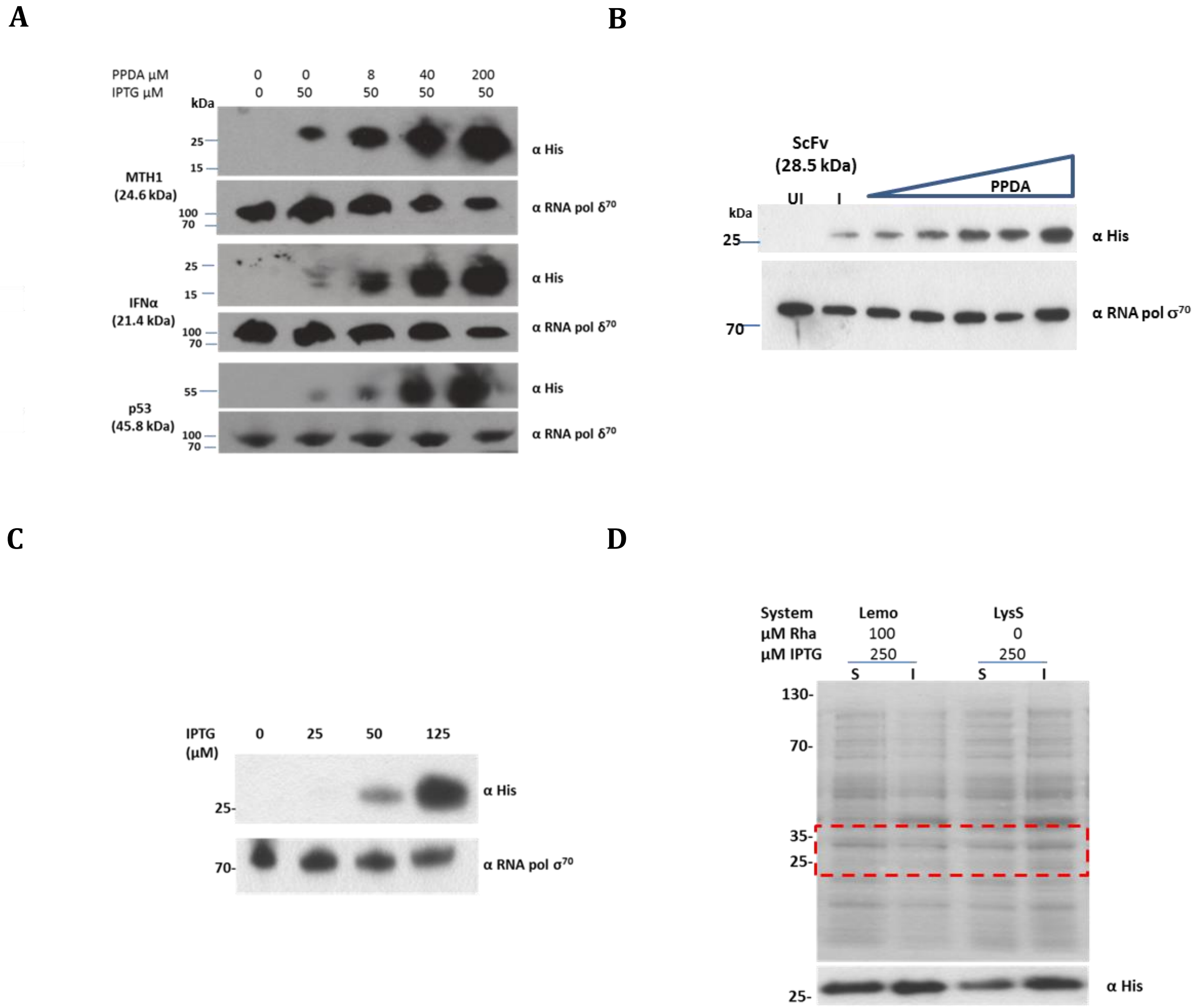

Supplementary Figure 10

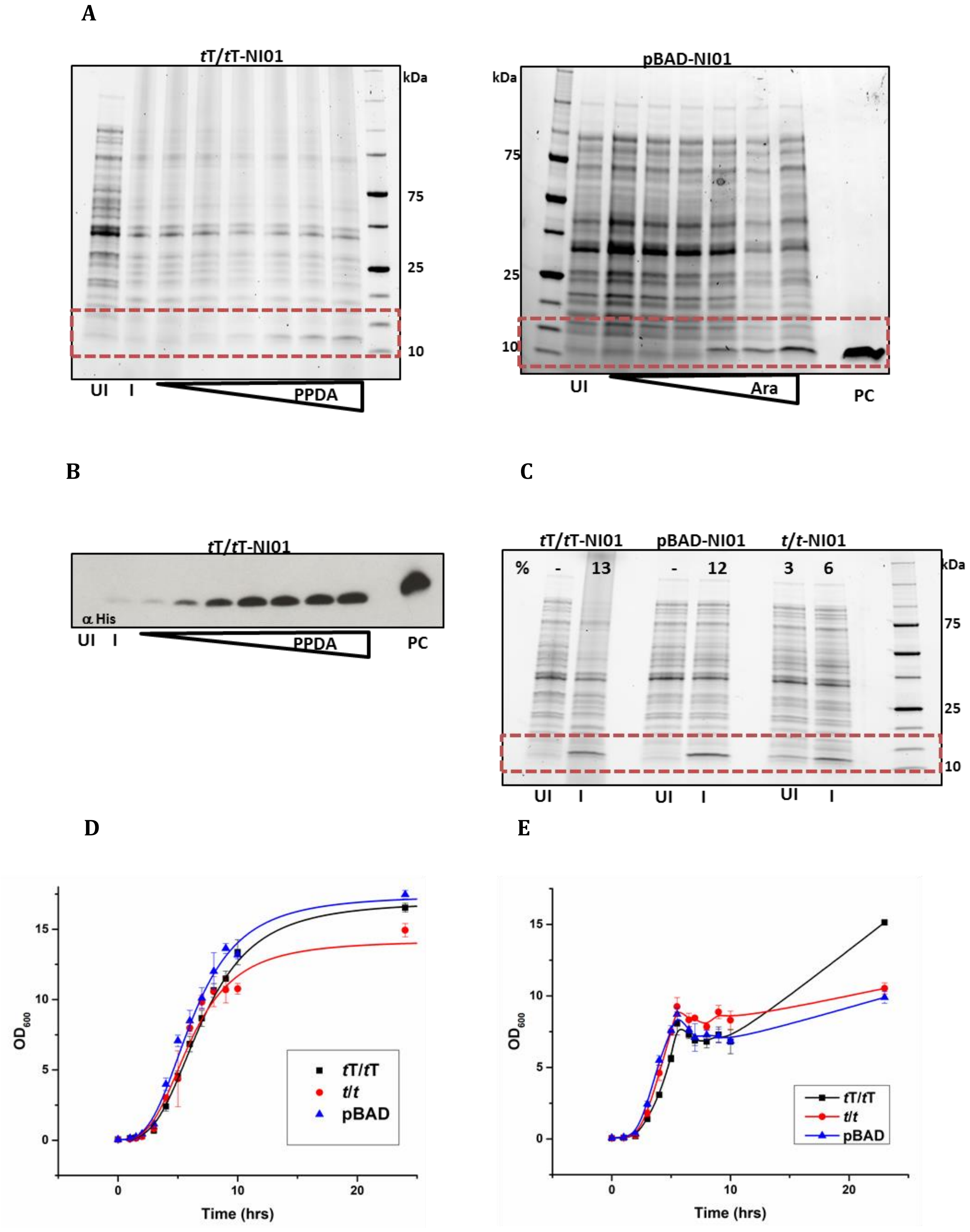

Supplementary Table 1

3 hrs induction at 30°C				
	t **/*t***	***t*** **/*t*** **T**	***t*** **T/*t***	***t*** **T/*t*** **T**
**Max**	130,157±4,199	87,128±1,352	148,463±5,488	89,053±1,9506
**Min**	2,054±173	286±2	358±74	105±1
**Fold increase**	65±8	305±3	428±83	845±12

20 hrs induction at 30°C				
	t **/*t***	***t*** **/*t*** **T**	***t*** **T/*t***	***t*** **T/*t*** **T**
**Max**	440,017±18,398	204,895±32,191	401,381±6,213	184,594±14,798
**Min**	175,509±5,206	24,264±3,820	7,438±2,034	555±9
**Fold increase**	3±0.18	8±0.01	56±10	333±32

Supplementary Table 2

***tT/t* system**

IPTG titration		IPTG (+ 40 μM PPDA)		IPTG (+ 200μM PPDA)		IPTG (+ 1000 μM PPDA)	
		value	std. error	value	std. error	value	std. error
min.	(RFU/OD)	547	32	1,326	51	3,229	1,021
max.	(RFU/OD)	108,778	6,176	135,051	3,977	148,490	2,685
x_0	(μM)	168.58	18.66	110.85	8.54	84.76	4.69
n_H		1.71	0.26	1.67	0.13	1.55	0.09
Reduced χ^2		2.87		1.16		1.56	
Adjusted R^2		0.99		1.00		1.00	
max./min.		198.98		101.82		45.98	
EC_{10}	(μM)	46.78		29.68		20.62	
EC_{50}	(μM)	168.58		110.85		84.76	
EC_{90}	(μM)	607.57		414.03		348.37	
**EC_{90}/EC_{10}**		**12.99**		**13.95**		**16.89**	

PPDA titration		PPDA (+ 40 μM IPTG)		PPDA (+ 200 μM IPTG)		PPDA (+ 1000 μM IPTG)	
		value	std. error	value	std. error	value	std. error
min.	(RFU/OD)	3,922	179	28,309	527	55,649	829
max.	(RFU/OD)	44,030	3,959	128,402	6,787	140,039	1,706
x0	(μM)	241.87	56.99	48.30	10.70	29.51	1.09
nH		1.00	0.11	1.16	0.12	1.12	0.08
Reduced χ2		0.02		0.29		0.02	
Adjusted R2		0.99		0.99		1.00	
max./min.		11.23		4.54		2.52	
EC_{10}	(μM)	27.16		7.28		4.14	
EC_{50}	(μM)	241.87		48.30		29.51	
EC_{90}	(μM)	2,154.15		320.46		210.10	
**EC_{90}/EC_{10}**		**79.32**		**44.01**		**50.69**	

***t/t* system**

		IPTG titration	
		value	std. error
min.	(RFU/OD)	2,349	931
max.	(RFU/OD)	145,835	5,695
x_0	(μM)	34.16	11.83
n_H		1.92	0.41
Reduced χ^2		168.87	
Adjusted R^2		0.99	
max./min.		62.07	
EC_{10}	(μM)	10.88	
EC_{50}	(μM)	34.16	
EC_{90}	(μM)	107.25	
**EC_{90}/EC_{10}**		**9.86**	

***tT/tT* system**

IPTG titration		IPTG (+ 40 μM PPDA)		IPTG (+ 200 μM PPDA)		IPTG (+ 1000 μM PPDA)	
		value	std. error	value	std. error	value	std. error
min.	(RFU/OD)	210.47	5.23	442.00	8.63	896.07	27.77
max.	(RFU/OD)	42,136.41	975.70	79,418.29	736.98	91,715.17	1,127.54
x_0	(μM)	148.66	8.15	136.24	2.99	86.80	4.81
n_H		1.52	0.05	1.44	0.02	1.42	0.04
Reduced χ^2		0.07		0.03		0.10	
Adjusted R^2		1.00		1.00		1.00	
max./min.		200.20		179.68		102.35	
EC_{10}	(μM)	35.03		29.51		18.51	
EC_{50}	(μM)	148.66		136.24		86.80	
EC_{90}	(μM)	630.87		629.12		407.03	
**EC_{90}/EC_{10}**		**18.01**		**21.32**		**21.99**	

PPDA titration		PPDA (+ 40 μM IPTG)		PPDA (+ 200 μM IPTG)		PPDA (+ 1000 μM IPTG)	
		value	std. error	value	std. error	value	std. error
min.	(RFU/OD)	2,139.63	221.03	5,178.51	166.06	8,906.38	187.05
max.	(RFU/OD)	39,517.75	12,075.69	78,124.07	2,491.40	94,440.87	1,779.80
x0	(μM)	586.68	484.14	114.67	11.05	65.87	4.98
nH		0.80	0.15	0.94	0.04	1.04	0.04
Reduced χ2		0.02		0.12		0.19	
Adjusted R2		0.99		1.00		1.00	
max./min.		18.47		15.09		10.60	
EC_{10}	(μM)	37.51		11.11		7.89	
EC_{50}	(μM)	586.68		114.67		65.87	
EC_{90}	(μM)	9,176.05		1,183.33		549.78	
**EC_{90}/EC_{10}**		**244.63**		**106.49**		**69.66**	

Supplementary Table 3

3 hrs inductions at 30°C						
	t /t	***t* T/*t* T**	**LysS**	**Lemo**	**KRX**	**pBAD**
**Max**	130,157±4,199	89,053±1,9506	141,119±4,579	175,429±10,152	72,353±4,791	123,201±3,096
**Min**	2,054±173	105±1	316±28	595±94	623±70	326±22
**Fold increase**	65±8	845±12	459±19	315±67	117±5	387±30

Supplementary Table 4

LysS system

		IPTG titration	
		value	std. error
min.	(RFU/OD)	289	98
max.	(RFU/OD)	135,091	12,825
x_0	(μM)	92.43	8.74
n_H		2.51	0.13
Reduced χ^2		5.91	
Adjusted R^2		0.99	
max./min.		467.30	
EC_{10}	(μM)	38.51	
EC_{50}	(μM)	92.43	
EC_{90}	(μM)	221.80	
**EC_{90}/EC_{10}**		**5.76**	

pBAD system

		Ara titration	
		value	std. error
min.	(RFU/OD)	327	41
max.	(RFU/OD)	126,917	5,568
x_0	(μM)	0.54	0.09
n_H		0.77	0.05
Reduced χ^2		0.59	
Adjusted R^2		1.00	
max./min.		388.49	
EC_{10}	(μM)	0.03	
EC_{50}	(μM)	277.21	
EC_{90}	(μM)	9.52	
**EC_{90}/EC_{10}**		**312.29**	

Lemo system

		IPTG (+ 100 μM Rha)		IPTG (+ 250 μM Rha)		IPTG (+ 1000 μM Rha)	
		value	std. error	value	std. error	value	std. error
min.	(RFU/OD)	828	97	657	63	601	60
max.	(RFU/OD)	119,337	10,199	107,224	12,471	110,379	32,352
x0	(μM)	88.66	15.47	130.55	28.08	304.21	123.24
nH		1.27	0.11	1.24	0.10	1.33	0.14
Reduced χ2		0.06		0.05		0.10	
Adjusted R2		1.00		0.99		0.99	
max./min.		144.09		163.10		183.55	
EC_{10}	(μM)	15.63		22.14		58.16	
EC_{50}	(μM)	88.66		130.55		304.21	
EC_{90}	(μM)	502.75		769.89		1,591.14	
**EC_{90}/EC_{10}**		**32.16**		**34.78**		**27.36**	

		Rha (+ 10 μM IPTG)		Rha (+ 50 μM IPTG)		Rha (+ 100 μM IPTG)	
		value	std. error	value	std. error	value	std. error
max.	(RFU/OD)	9,045	842	51,352	8,834	67,783	8,786
min.	(RFU/OD)	1,213	47	5,696	693	12,013	716
x0	(μM)	213.62	31.22	253.94	71.70	310.83	72.48
nH		1.83	0.20	1.66	0.32	1.84	0.37
Reduced χ2		0.00		0.05		0.13	
Adjusted R2		0.99		0.98		0.98	
max./min.		7.46		9.02		5.64	
EC_{10}	(μM)	64.44		67.57		94.07	
EC_{50}	(μM)	213.62		253.94		310.83	
EC_{90}	(μM)	708.12		954.43		1,027.09	
**EC_{90}/EC_{10}**		**10.99**		**14.13**		**10.92**	

KRX system

		Rha (+ 50 μM IPTG)		Rha (+ 250 μM IPTG)		Rha (+ 500 μM IPTG)	
		value	std. error	value	std. error	value	std. error
min.	(RFU/OD)	1,162	42	1,183	68	1,249	30
max.	(RFU/OD)	74,675	3,894	85,719	7,373	80,017	2,686
x0	(μM)	639.46	106.96	911.21	229.57	677.43	62.74
nH		0.96	0.06	0.92	0.08	0.92	0.04
Reduced χ2		0.04		0.10		0.01	
Adjusted R2		1.00		1.00		1.00	
max./min.		64.28		72.45		64.08	
EC_{10}	(μM)	64.90		84.30		62.70	
EC_{50}	(μM)	639.46		911.21		677.43	
EC_{90}	(μM)	6,300.47		9,848.89		7,319.08	
**EC_{90}/EC_{10}**		**97.08**		**116.83**		**116.73**	

Supplementary Table 5

T°C	hrs	System	Max RFU/OD	Basal RFU/OD	Basal (% of max)	Max/Basal	Vol yield (mg/L)	OD_{600}
20	5	*t* /*t*	297,883±32,903	1,123±13	0.38	265±26	231±13	2±0.1
		t T/*t* T	69,078±4,373	187±0.84	0.27	369±25	86±0.4	3±0.3
30	5	*t* /*t*	321,131±17,212	11,457±831	3.57	28±2	740±37	6±1
		t T/*t* T	101,535±160	204±16	0.20	500±38	395±3.5	9±0.5
		LysS	156,454±2,284	3,942±142	2.52	42±3	913±29	11±1.5
		Lemo	166,478±2,812	3,768±191	2.26	44±3	601±22	7±1.0
		KRX	55,306±1,311	632±18	1.14	84±2	310±24	10±0.1
		pBAD	115,755±4,801	339±2	0.29	345±26	280±3	12±0.6
37	5	*t* /*t*	135,655±3,149	10,816±96	7.97	12±0.20	244±4	5±0.5
		t T/*t* T	34,264±411	158±2	0.46	216±0.71	147±0.8	9±1.5

T°C	hrs	System	Max RFU/OD	Basal RFU/OD	% basal	Fold increase	Vol yield (mg/L)	OD_{600}
20	20	*t* /*t*	297,541±4,376	36,235±1,018	12.18	9±0.7	2,950±57	25±1
		t T/*t* T	220,791±2,202	1,096±6	0.50	201±0.95	2,024±17	28±1
30	20	*t* /*t*	44,264±7,770	155,309±10,247	351	0.28±0.06	466±37	21±2
		t T/*t* T	267,577±925	633±1	0.24	423±0.61	1,579±2	21±3
		LysS	99,325±498	15,471±1,200	15.58	7±1	915±23	17±2
		Lemo	80,139±4,772	3,881±288	4.84	22±5	460±63	13±2*
		KRX	130,478±1,804	2,337±37	1.79	55±2	461±23	9.3±0.4*
		pBAD	104,834±3,720	694±16	0.62	152±10	260±7	23±1
37	20	*t* /*t*	2,442±60	122,247±7,865	5,015	0	32±0.22	18±2
		t T/*t* T	151,050±870	352±2	0.23	429±0.19	859±7	22±3

* half value of the UI samples

Supplementary Table 6

T°C	Time (hrs)	System	Inducers	Max RFU/OD	DbT	Specific rate RFU/OD/hrs	Volumetric rate mg/L/hrs
20	5	*t* /*t*	–	1,451±108	3.86±0.15		
			+	257,222±36,699	4.90±0.06	53,731±6,557	52±3
30	5	*t* /*t*	–	6,732±939	2.24±0.29		
			+	458,103±13,502	4.70±0.13	102,472±4,925	312±4
37	5	*t* /*t*	–	28,590±5,340	1.94±0.01		
			+	188,387±25,718	4.90±0.13	48,687±3,714	147±10
20	5	*t* T/*t* T	–	494±27	3.82±0.73		
			+	135,345±16,634	4.14±0.97	27,439±4,232	63±2
30	5	*t* T/*t* T	–	286±8	0.85±0.03		
			+	108,052±1,166	0.99±0.11	22,293±3,546	71±3
37	5	*t* T/*t* T	–	197±8	1.51±0.09		
			+	92,870±243	1.40±0.21	20,962±1,072	77±10

Supplementary Table 7

t/*t* system

IPTG titration		20°C value	20°C std. error	30°C value	30°C std. error	37°C value	37°C std. error
minimum	(RFU/OD/hrs)	604	224	982	187	759	603
maximum	(RFU/OD/hrs)	58,968	3,271	118,085	10,510	33,379	644
**% max**		**1.02**		**0.83**		**2.27**	
x_0	(μM)	71.59	6.37	48.68	6.72	6.78	0.5
n_H		1.99	0.15	1.31	0.09	1.81	0.22
Reduced χ^2		0.05		0.92		651,698.04	
Adjusted R^2		1.00		1.00		1.00	
**EC_{90}/EC_{10}**		**9.10**		**28.73**		**11.30**	

*t*T/*t*T system

PPDA titration		20°C value	20°C std. error	30°C value	30°C std. error	37°C value	37°C std. error
minimum	(RFU/OD/hrs)	2,782	86	2,933	137	2,201	672
maximum	(RFU/OD/hrs)	26,303	1,017	23,279	1,417	23,605	3,311
**% max**		**10.58**		**12.60**		**9.32**	
x_0	(μM)	19.00	2.81	29.45	5.59	69.34	22.7
n_H		1.01	0.06	1.00	0.08	1.01	0.26
Reduced χ^2		0.10		0.02		0.60	
Adjusted R^2		1.00		1.00		1.00	
**EC_{90}/EC_{10}**		**76.90**		**80.99**		**77.10**	

IPTG titration		30°C value	30°C std. error	37°C value	37°C std. error
minimum	(RFU/OD/hrs)	87	68	2	0
maximum	(RFU/OD/hrs)	12,777	446	10,502	2,706
**% max**		**0.68**		**0.02**	
x_0	(μM)	35.49	2.63	171.18	75.46
n_H		1.12	0.06	0.89	0.05
Reduced χ^2		9,275.69		0.40	
Adjusted R^2		1.00		1.00	
**EC_{90}/EC_{10}**		**50.33**		**138.09**	

Supplementary Table 8

A

IPTG Concentration	Events	Mean	Median	CV
**0uM**	100000 (100%)	52013.57	43585.92	70.59
	**Low Peak (3.79%)**	**315.49**	**296.8**	**37.93**
	**High Peak (95.62%)**	**54139.13**	**45120.88**	**64.93**
**10uM**	100000 (100%)	188813.12	216237.84	64.28
	**Low Peak (19.96%)**	**383.54**	**352.8**	**45.65**
	**High Peak (73.37%)**	**248323.06**	**242894.41**	**26.76**
**25uM**	100000 (100%)	128223.72	797.44	126.80
	**Low Peak (55.41%)**	**499.24**	**365.12**	**105.80**
	**High Peak (41.64%)**	**304869.18**	**301375.2**	**31.05**
**50uM**	100000 (100%)	103476.66	488.32	169.39
	**Low Peak (67.02%)**	**452.68**	**365.12**	**79**
	**High Peak (27.89%)**	**364225.52**	**365736**	**32.92**
**125uM**	100000 (100%)	25666.69	369.60	410.12
	**Low Peak (90.12%)**	**372.9**	**352.8**	**44.55**
	**High Peak (5.66%)**	**410707.52**	**401406.33**	**40.95**
**250uM**	100000 (100%)	6767.72	328.16	781.68
	**Single Peak (96.31%)**	**351.34**	**323.68**	**52.16**

B

IPTG Concentration	Events	Mean	Median	CV
**Uninduced**	100000 (100%)	474.99	445.76	48.43
	**Single Peak (99.74%)**	**472.8**	**446.88**	**39.33**
**0.013 uM**	100000 (100%)	4596.96	1519.84	138.07
	**Single Peak (99.85%)**	**4578.8**	**1522.08**	**136.43**
**0.130 uM**	100000 (100%)	16085.75	14632.24	60.32
	**Single Peak (99.18%)**	**16164.3**	**14700**	**58.77**
**1.300 uM**	100000 (100%)	32020.19	28953.12	49.38
	**Single Peak (98.5%)**	**32336.49**	**29162.56**	**44.52**
**13.30 uM**	100000 (100%)	41426.21	38968.16	35.06
	**Single Peak (98.5%)**	**41586.37**	**39072.32**	**30.3**
**133 uM**	100000 (100%)	74407.59	71851.36	32.56
	**Single Peak (98.68%)**	**75126.58**	**72159.92**	**30**
**330 uM**	100000 (100%)	90225.81	86048.48	30.83
	**Single Peak (99.04%)**	**90805.88**	**86282.56**	**28.77**

C

IPTG Concentration	Events	Mean	Median	CV
**0uM**	100000 (100%)	283.2	269.92	64.38
	**Single Peak (99.12%)**	**283.62**	**269.92**	**38.80**
**10uM**	100000 (100%)	5411.10	5073.6	48.76
	**Low Peak (3.08%)**	**296.33**	**276.64**	**33.6**
	**High Peak (95.43%)**	**5619.28**	**5203.52**	**42.78**
**25uM**	100000 (100%)	11182.53	10731.28	44.74
	**Low Peak (2.86%)**	**345.92**	**322.92**	**32.93**
	**High Peak (95.38%)**	**11644.92**	**10972.64**	**38.25**
**50uM**	100000 (100%)	16176.72	15757.28	42.67
	**Low Peak (3.29%)**	**367.46**	**342.72**	**36.01**
	**High Peak (94.56%)**	**16966.72**	**16159.36**	**34.75**
**125uM**	100000 (100%)	25204.51	25010.72	43.86
	**Low Peak (4.22%)**	**542.23**	**508.48**	**31.28**
	**High Peak (92.38%)**	**26946.43**	**25794.72**	**32.44**
**250uM**	100000 (100%)	28891.77	28949.76	42.21
	**Gated (92.34%)**	**30873.67**	**29795.36**	**30.44**

D

Induction condition	Events	Mean	Median	CV
**Uninduced**	100000 (100%)	325.10	305.76	46.38
	**Single Peak (99.6%)**	**324.65**	**306.88**	**41.12**
**IPTG (50uM)**	100000 (100%)	4729.36	4527.04	33.81
	**Single Peak (99.98%)**	**4737.31**	**4536**	**29.15**
**1.6uM PPDA**	100000 (100%)	10945.52	10520.16	30.44
	**Single Peak (98.99%)**	**10996.69**	**10543.68**	**27.68**
**8uM PPDA**	100000 (100%)	20363.6	19682.88	28.38
	**Single Peak (98.85%)**	**20476.9**	**19727.68**	**25.11**
**40uM PPDA**	100000 (100%)	34763	34144.32	29.95
	**Single Peak (96.89%)**	**35562.63**	**34387.36**	**23.26**
**92uM PPDA**	100000 (100%)	43601.67	43380.96	37.13
	**Low Peak (4.41%)**	**241.65**	**231.84**	**33.24**
	**High Peak (93.41%)**	**45980.13**	**44059.68**	**24.64**
**200uM PPDA**	100000 (100%)	49597.30	49862.40	36.38
	**Low Peak (5.37%)**	**239.91**	**227.36**	**35.99**
	**High Peak (93.52%)**	**52785.81**	**50780.24**	**23.68**
**400uM PPDA**	100000 (100%)	53761.14	55675.76	55.55
	**Low Peak (9.57%)**	**246.21**	**232.96**	**38.07**
	**High Peak (88.79%)**	**59674.16**	**57632.96**	**25.53**
